# A new genome allows the identification of genes associated with natural variation in aluminium tolerance in *Brachiaria* grasses

**DOI:** 10.1093/jxb/eraa469

**Published:** 2020-10-16

**Authors:** Margaret Worthington, Juan Guillermo Perez, Saule Mussurova, Alexander Silva-Cordoba, Valheria Castiblanco, Juan Andres Cardoso Arango, Charlotte Jones, Narcis Fernandez-Fuentes, Leif Skot, Sarah Dyer, Joe Tohme, Federica Di Palma, Jacobo Arango, Ian Armstead, Jose J De Vega

**Affiliations:** 1 International Center for Tropical Agriculture (CIAT), Cali, Colombia; 2 Earlham Institute, Norwich Research Park, Norwich, UK; 3 Institute of Biological, Environmental and Rural Sciences (IBERS), Aberystwyth University, Aberystwyth, UK; 4 Pontificia Universidad Católica de Chile, Chile

**Keywords:** Acid soils, aluminium tolerance, *Brachiaria*, differential expression, genome assembly, grass, QTL mapping, *Urochloa*

## Abstract

Toxic concentrations of aluminium cations and low phosphorus availability are the main yield-limiting factors in acidic soils, which represent half of the potentially available arable land. *Brachiaria* grasses, which are commonly sown as forage in the tropics because of their resilience and low demand for nutrients, show greater tolerance to high concentrations of aluminium cations (Al^3+^) than most other grass crops. In this work, we explored the natural variation in tolerance to Al^3+^ between high and low tolerant *Brachiaria* species and characterized their transcriptional differences during stress. We identified three QTLs (quantitative trait loci) associated with root vigour during Al^3+^ stress in their hybrid progeny. By integrating these results with a new *Brachiaria* reference genome, we identified 30 genes putatively responsible for Al^3+^ tolerance in *Brachiaria*. We observed differential expression during stress of genes involved in RNA translation, response signalling, cell wall composition, and vesicle location homologous to aluminium-induced proteins involved in limiting uptake or localizing the toxin. However, there was limited regulation of malate transporters in *Brachiaria*, which suggests that exudation of organic acids and other external tolerance mechanisms, common in other grasses, might not be relevant in *Brachiaria*. The contrasting regulation of RNA translation and response signalling suggests that response timing is critical in high Al^3+^-tolerant *Brachiaria*.

## Teaser

We identified QTLs, genes, and molecular responses in high and low tolerant *Brachiaria* grasses associated with aspects of the response to aluminium stress, such as regulation, cell wall composition, and active transport.

## Introduction

Acidic soils constitute ~30% of the world’s total land area and 50% of the potentially available arable land (Von Uexküll and Mutert, 1995). Acidic soils are particularly predominant in a northern ‘temperate belt’ and a southern ‘subtropical belt’. Therefore, a broad range of vegetable, cereal, and forage crops can be yield limited in these conditions (Hede *et al.*, 2001). The adverse effects of soil acidity are mostly associated with several mineral toxicities and deficiencies, particularly increased concentrations of soluble forms of manganese, iron, and aluminium, and reduced levels of available forms of phosphorus (P), calcium, magnesium, and potassium. Among these, lower P solubility and aluminium toxicity are considered the main limiting factors on productivity (Eswaran *et al.*, 1997; Kochian *et al.*, 2015). As soil pH decreases below 5, aluminium becomes soluble as the aluminium trivalent cation (Al^3+^), a form highly toxic to plants. Soluble Al^3+^ effect on root apices results in diminished ion and water uptake.

Although acid soils can be conditioned for improved agricultural use through the addition of lime, in general, the most sustainable strategy is a combination of agronomic practices and growing tolerant cultivars. Natural variation in aluminium tolerance has been identified for several crops, with rice (*Oryza sativa*) being the most aluminium tolerant among the food staples (Rao *et al.*, 2016). For crops to maintain growth on acid soils, they need to be adaptive to toxic levels of H^+^ (low pH), Al^3+^ toxicity, and P deficiency (Kochian *et al.*, 2015; Rao *et al.*, 2016). Furthermore, since pH, levels of rhizotoxic Al^3+^ species, and P availability are not homogeneously distributed in soil layers, plant roots have to deal with different levels of these factors during their growth (Liao *et al.*, 2006; Liang *et al.*, 2013) by regulating the available genetic repertoire during the growing season. Several root architectural, morphological, anatomical, and metabolic factors associated with yield on acidic soils were reviewed by Rao *et al.* (2016). Putative cellular mechanisms for plant adaptation to low pH stress are diverse, including changes in organic acid metabolism in the cytoplasm (Martínez-Camacho *et al.*, 2004), changes in the pectic polysaccharide network in the cell wall (Koyama *et al.*, 2001), or regulation of root cell membrane transporters mediating chelator efflux (Liang *et al.*, 2013).

All *Brachiaria* (Trin.) Griseb. (syn. *Urochloa* P. Beauv.) species show greater tolerance to Al^3+^ toxicity than most other grass crops, including maize (*Zea mays*), rice, or wheat (*Triticum aestivum*) (Kochian *et al.*, 2015). *Brachiaria* grasses are native to East Africa and are widely sown as forage to feed ruminants on acid tropical soils across the tropics, particularly in areas with marginal soils. *Brachiaria* has a set of desirable genetic characteristics linked to drought and waterlogging tolerance, tolerance of poor and acidic soils, and resistance to major diseases (Rao *et al.*, 2016). However, *Brachiaria* species have a low demand for soil nutrients (Pizarro *et al.*, 2013). Toxic cation levels (and not reduced levels of mineral solutes) are the most limiting factors for their productivity under acidic soil conditions (Rao *et al.*, 1996). In *Brachiaria* grasses, there is considerable variation in Al^3+^ tolerance; *Brachiaria decumbens* is significantly more tolerant to Al^3+^ than *B. ruziziensis* (Arroyave *et al.*, 2011; Bitencourt *et al.*, 2011). In general, Al^3+^ tolerance mechanisms are classified as external and internal tolerance mechanisms; the molecular genetic mechanisms underlying the stress-induced exudation of organic acids are well known (Kochian *et al.*, 2015). In *Brachiaria*, Al^3+^ tolerance mechanism responses have been mostly associated with the exclusion of Al^3+^ (external tolerance mechanisms): studies comparing the responses of tolerant *B. decumbens* and sensitive *B. ruziziensis* under Al^3+^ stress showed that *B. decumbens* exhibited a multiseriate root exodermis and aluminium accumulation in root hairs (Arroyave *et al.*, 2011), and downgraded the importance of exudation of organic acids or changes in rhizosphere pH (Wenzl *et al.*, 2001). Internal tolerance mechanisms remain largely unknown in *Brachiaria.*

Although the studies mentioned above provide some insight into the mechanisms of Al^3+^ tolerance in *Brachiaria*, little is known about its genetic regulation. Significant advances have been made in defining the mechanisms of Al^3+^ resistance in different crops and forages using cultivars resistant and sensitive to this stress; screening experiments have revealed different but consistent aluminium tolerance among *Brachiaria* species and cultivars, which indicates the genetic basis of the trait (Arroyave *et al.*, 2013). The three most important commercial species, *Brachiaria brizantha* (A. Rich.) Stapf., *B. decumbens* Stapf., and *B. humidicola* (Rendle) Schweick, exist primarily as apomicts with varying levels of polyploidy (Valle and Savidan, 1996). The diploid sexual species *B. ruziziensis* (Germ. & C.M. Evrard) is also used in breeding as a bridge between apomictic species (Miles, 2007).

*Brachiaria* is one of the most widely used forage genera in the American tropics and a promising one for sub-Saharan Africa. For the potential of these genera to be realized, varieties must be tailored to the particular demands of each environment in which they are grown (Bailey-Serres *et al.*, 2019). While genomic approaches can accelerate genetic gain from crop breeding, these approaches rely on resources that are costly and often scarce in ‘orphan crops’ such as *Brachiaria*. Here, we have produced fundamental genomic resources previously absent for *Brachiaria*, including a reference genome.

Tolerant plants need to deal with the heterozygous soil layers with variable toxic concentrations. Plants do this by regulating their available genetic repertoire, among other options. The aim of this study is developing a better understanding of the molecular regulation of Al^3+^ tolerance, a critical component affecting *Brachiaria* productivity in acid soils. For that, we will use forward genetics and newly produced genomic resources and the differential tolerance among *Brachiaria* species.

## Materials and methods

### Plant materials and phenotyping

The interspecific mapping population used in this work consisted of 169 genotypes of F_1_ progeny from a cross between the synthetic autopolyploid *B. ruziziensis* accession BRX 44-02 and the segmental allopolyploid *B. decumbens* accession CIAT 606 (cv. Basilisk). This population was generated initially to create saturated linkage maps and identify markers linked to apomixis (Worthington *et al.*, 2016). Accessions were phenotyped for aluminium tolerance at CIAT in Cali, Colombia, following Wenzl *et al.* (2006). Briefly, the experiment consisted of six cycles (replications over time) with 1–7 replicate plants per cycle. Plants were grown for 20 d in hydroponic solutions with 0 or 200 μM AlCl_3_ and phenotyped in control and Al^3+^ stress conditions for cumulative root length, root biomass, and average root diameter at the end of each experimental cycle. Data were transformed to meet the assumption of normality; root length and biomass were square-root transformed, and root diameter was natural log-transformed. The PROC MIXED method (SAS v. 9.2, Cary, NC, USA) was used to fit a random effect model. Genotypic best linear unbiased predictors (BLUPs) were calculated from stress (200 μM AlCl_3_) and control (0 μM AlCl_3_) conditions individually and back-transformed to calculate the relative root length (RRL), relative root biomass (RRB), and relative root diameter (RRD) ratios between stress and control (stress/control).

### Genome sequencing, assembly, and annotation

We selected the *B. ruziziensis* genotype CIAT 26162 (2*n*=2*x*=18) as the source of genomic DNA. This is a semi-erect diploid accession from Burundi (–3.1167, 30.1333) that was probably mutagenized with colchicine to produce the synthetic autotetraploid *B. ruziziensis* BRX 44-02, one of the progenitors of the interspecific mapping population analysed. The ploidy of CIAT 26162 has recently been verified by cytogenetics (P. Tomaszewska, personal communication). Two paired-end libraries were created and sequenced in Illumina HiSeq 2500 machines in rapid run mode by the Earlham Institute (~70×) and the Yale Center for Genome Analysis (~30×). Additionally, a Nextera mate-pair (MP) library with an insert length of 7 kb was sequenced to improve the scaffolding. Read quality was assessed, and contaminants and adaptors removed. Illumina Nextera MP reads were required to include a fragment of the adaptor to be used in the following steps (Leggett *et al.*, 2013a). The paired-end shotgun libraries were assembled and later scaffolded using the MP library with Platanus v1.2.117, which is optimized for heterozygous genomes (Kajitani *et al.*, 2014). We did not use Platanus’ gap-closing step. Approximately 1 million Pacbio reads from this same genotype were generated in a PacBio RSII sequencer and used for gap filling using PBJelly v.15.8.24 (English *et al.*, 2012). Scaffolds shorter than 1 kbp were filtered out. We used 31mer spectra analysis to compare the assemblies produced by different pipelines, as well as our final assembly with the assemblies from preceding steps. A k-mer spectrum is a representation of how many fixed-length words or k-mers (*y*-axis) appear a certain number of times or coverage (*x*-axis). The k-mer counting was performed with KAT (Mapleson *et al.*, 2016). The completeness of the assembly was checked with BUSCO (Simão *et al.*, 2015).

Repetitive and low complexity regions of the scaffolds were masked using RepeatMasker (Tarailo-Graovac and Chen, 2009) based on self-alignments and homology with the RepBase public database and specific databases built with RepeatModeler (Smit and Hubley, 2008). Long terminal repeat (LTR) retrotransposons were detected by LTRharvest (Ellinghaus *et al.*, 2008) and classified with RepeatClassifier. The 5' and 3′ ends of each LTR were aligned to each other with MUSCLE (Edgar, 2004) and used to calculate the nucleotide divergence rate with the Kimura-2 parameter using MEGA6 (Tamura *et al.*, 2013). The insertion time was estimated by assuming an average substitution rate of 1.3×10^–8^ (Schmutz *et al.*, 2014)

Our annotation pipeline (De Vega *et al.*, 2015) uses four sources of evidence: (i) *de novo* and genome-guided *ab initio* transcripts deduced from RNA-seq reads from the *B. ruziziensis* genotype assembled with Trinity (Grabherr *et al.*, 2011), and Tophat and Cufflinks (Trapnell *et al.*, 2012); (ii) gene models predicted by Augustus (Stanke *et al.*, 2006); (iii) homology-based alignments of transcripts and proteins from four close species with Exonerate and GMAP (Wu *et al.*, 2016); and (iv) the repeats annotation. Finally, MIKADO (Venturini *et al.*, 2018) built the gene models to be compatible with this previous information. Proteins were compared with the NCBI non-redundant proteins and EBI’s InterPro databases, and the results were imported into Blast2GO (Conesa *et al.*, 2005) to annotate the Gene Ontology (GO) and GO slim terms, enzymatic protein codes, and KEGG (Kyoto Encyclopedia of Genes and Genomes) pathways. Proteins were also functionally annotated with the GO terms of any significant orthologous protein in the eggNOG database (Powell *et al.*, 2014), using the eggNOG-mapper pipeline (Huerta-Cepas *et al.*, 2017).

### Comparative genomics

Syntenic blocks between *B. ruziziensis* and foxtail millet [*Setaria italica* (L.) P. Beauv] whole genomes were identified with Minimap (Li, 2016), and plotted with D-GENIES (Cabanettes and Klopp, 2018). Previously, we filtered out any scaffold <10 kbp that did not contain any gene. The assembly was anchored to foxtail millet by assigning each scaffold to the chromosome position where it had the longest alignment chain after combining proximal alignments. For clustering, proteins from five related species were assigned to eggNOG orthologous groups as before. A phylogenetic tree based on these data was built with MUSCLE by aligning the orthologous proteins within each eggnog cluster, filtering sets with one member per species (*Panicum virgatum* was excluded from this analysis), and finally estimating the nucleotide divergence rate using MEGA v6, as before.

### Population genotyping and genetic map construction

Genotyping-by-sequencing (GBS) libraries were prepared and sequenced for the 169 F_1_ progenies and the two parents as described in Worthington *et al.* (2016). Reads were demultiplexed according to the forward and reverse barcodes used during library preparation with FastGBS (Torkamaneh *et al.*, 2017), and adaptors and enzymatic motifs were removed with Cutadapt (Martin, 2011). Reads were aligned to the genome using BWA MEM (Li, 2013, Preprint). Single nucleotide polymorphism (SNP) calling was done for each sample with GATK’s Haplotycaller (Van der Auwera *et al.*, 2013) without the duplicated read filter (-drf DuplicateRead) and recalled for the population with GATK’s GenotypeGVCFs, in both tools with ‘--ploidy 4’. In agreement with filtering criteria previously tested in tetraploid *Brachiaria* samples (Worthington *et al.*, 2016), we used SNPs only, required at least 12 reads to call a homozygous site in any sample, and a minimum allele frequency of 5%. We removed any site not called in a progenitor or in >20% of the progeny. Markers that were heterozygous in only one parent and had a segregation ratio of a heterozygote to homozygote progeny of ~1:1 (between 0.5 and 1.75) were classified as single-dose allele (SDA) markers and used in the linkage map construction. Separate genetic linkage maps of BRX 44-02 and CIAT 606 were constructed in JoinMap v5 (Van Ooijen, 2011) using a threshold linkage logarithm of odds (LOD) score of 10 to establish linkage groups. Marker order was then determined using regression mapping with default settings. Downstream quantitative trait locus (QTL) analysis with MapQTL 6 (Van Ooijen, 2009) was conducted using only the *B. decumbens* CIAT 606 map.

### RNA-seq sequencing and analysis

Two replicate plants of *B. decumbens* CIAT 606 (cv. Basilisk) and *B. ruziziensis* BRX 44-02 were grown in high aluminium (200 μM AlCl_3_) and control (0 μM AlCl_3_) conditions in the greenhouse, as described previously. After 3 d of growth, roots and leaves were harvested from each plant. RNA extraction was performed with the RNeasy Plant Mini kit (Qiagen, CA, USA) and sent to the sequencing service provider (Earlham Institute, Norwich, UK) where Illumina RNA-seq libraries were prepared and sequenced using the HiSeq 2500 platform. Sixteen libraries were independently generated and sequenced: two tissues (roots and leaves), from two species (*B. ruziziensis* and *B. decumbens*), in two conditions (0 and 200 μM AlCl_3_ hydroponic solutions), and two replicates. Contaminations in the raw data were discarded with Kontaminant (Leggett *et al.*, 2013b). Adaptors were removed with Cutadapt (Martin, 2011) and quality checked with FastQC (Andrews, 2017). Reads were mapped to the assembled genome using STAR (Dobin *et al.*, 2013) and the gene models annotated for guidance. Counts were estimated with Stringtie (Pertea *et al.*, 2015). We used DEseq v2 (Love *et al.*, 2014) for analysing differential expression. Enriched GO terms and other categories in each group of differentially expressed genes (DEGs)were identified in R using TOPGO (Alexa and Rahnenfuhrer, 2010) employing a Fisher’s test [false discovery rate (FDR) <0.05] and the ‘weight01’ algorithm. The relationship among GO terms was plotted in R using ggplot (Wickham and Chang, 2008). We also reanalysed using this pipeline the publicly available data (PRJNA314352) from Salgado *et al.* (2017), obtained from harvested root tips from 12-day-old *B. decumbens* cv. Basilisk seedlings screened in similar experimental conditions to those described in our study but after 8 h of treatment (instead of 72 h).

## Results

### Root morphology in *Brachiaria* species in different aluminium cation concentrations

**Fig. 1.**
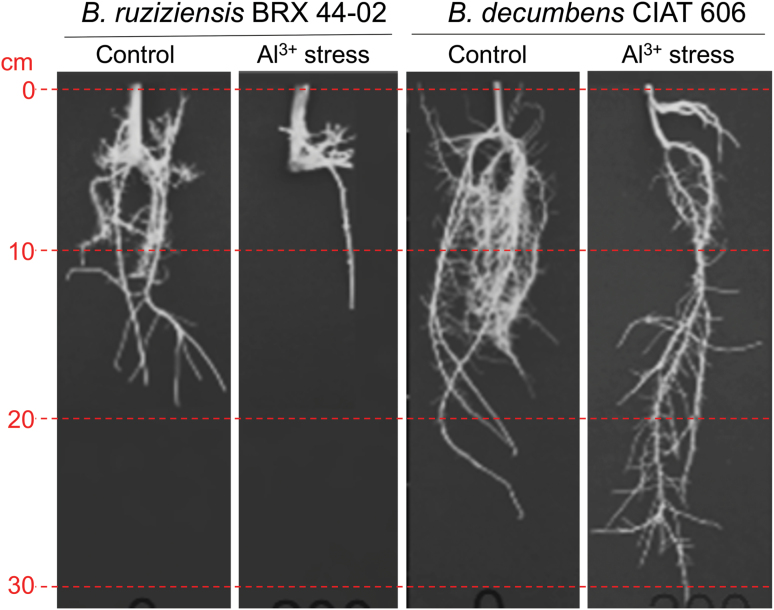
Root growth in *B. decumbens* accession CIAT 606 (cv. Basilisk) and *B. ruziziensis* accession BRX 44-02 after growing for 20 d in hydroponic solutions containing control (0 μM) and high (200 μM) Al^3+^ concentrations.

We demonstrated the superior aluminium tolerance of the *B. decumbens* accession CIAT 606 compared with *B. ruziziensis* accession BRX 44-02 (Fig. 1). The root morphology of CIAT 606 was less affected than that of BRX 44-02 after growing for 20 d in high aluminium (200 μM AlCl_3_) and control (0 μM AlCl_3_) concentration hydroponic solutions. Under stress conditions, the roots of *B. decumbens* were over three times as long and had twice the biomass of *B. ruziziensis.* However, the root diameter increased in stress conditions in a similar ratio in both species (see Supplementary Table S1 at *JXB* online).

**Fig. 2.**
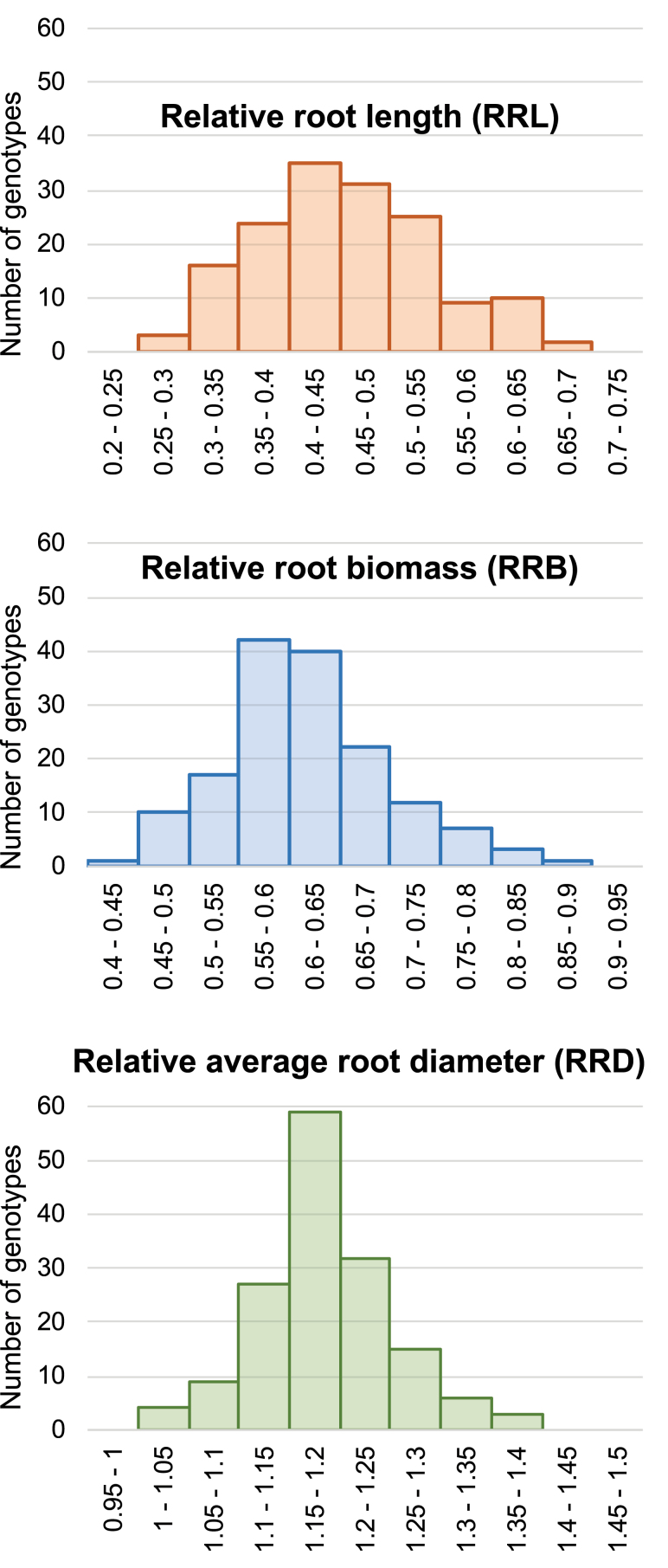
Relative root length, relative root biomass, and relative average root diameter ratios between Al^3+^ stress and control conditions (stress/control) in the interspecific progeny (*n*=169) between *B. ruziziensis* BRX 44-02 and *B. decumbens* CIAT 606 (cv. Basilisk).

The interspecific progeny obtained by crossing aluminium-tolerant *B. decumbens* accession CIAT 606 and aluminium-sensitive *B. ruziziensis* accession BRX 44-02 showed segregation in RRL, RRB, and RRD ratios (Fig. 2; Supplementary File 1 available at the Zenodo repository http://dx.doi.org/10.5281/zenodo.3941963). We obtained highly significant (*P*<0.001) genotypic differences in control and stress conditions for the three traits (root length, biomass, and diameter). The root biomass (*r*=0.73), length (*r*=0.76), and diameter (*r=*0.69) of the progeny were significantly correlated when grown in stress (200 μM AlCl_3_) and non-stress (0 μM AlCl_3_) conditions.

### Assembly and annotation of a *Brachiaria* reference genome

A whole-genome assembly (WGA) of CIAT 26162 was produced using Platanus v.1.2.4 (Kajitani *et al.*, 2014), from Illumina paired-end and Nextera MP reads with a coverage of ~100× and 7×, respectively (Supplementary Table S2). Platanus outperformed the contiguity results obtained with other pipelines. Although the combination of ABySS for the contig assembly and SOAP2 for the scaffolding resulted in a larger assembly, a k-mer frequency analysis (Mapleson *et al.*, 2016) evidenced that the additional content was repeated undercollapsed heterozygosity that Platanus had purged instead (Supplementary Fig. S1). A gap-filling step using ~1 million Pacbio reads with an average length of 4.8 kbp resulted in a reduced percentage of ambiguous nucleotides (Ns) from 17.45% to 11.39%. We finally discarded all the sequences <1 kbp to produce the reference genome we used for the downstream analysis (Table 1). To assess the completeness of the assembly, we verified that 1345 (93.4 %) of the 1440 BUSCO orthologous genes (Simão *et al.*, 2015) were present in the assembly, 1216 of which were completed; and in a single copy, 32 were duplicated, and 97 were fragmented. This WGA is deposited at NCBI with the accession number GCA_003016355. The raw reads are deposited in the Bioproject PRJNA437375.

**Table 1.** Statistics associated with the assembly of the *Brachiaria ruziziensis* reference genome and anchoring in pseudo-molecules

	Whole-genome assembly (WGA)	Pruned WGA^*a*^	Anchored assembly
**Total length**	732.5 Mbp	533.9 Mbp	525.1 Mbp
**% Ns**	10.6%	11.7%	12.18%
**Number of sequences**	102 579	23 076	9 pseudo-Chrs
**N50**	27.8 kbp	44.6 kbp	55.9 Mbp
**N20**	76.1 kbp	91.7 kbp	62.7 Mbp
**N80**	3.9 kbp	19.5 kbp	44.1 Mbp

The WGA is deposited in NCBI, accession number GCA_003016355. Anchoring is available in AGP format, together with the raw reads, in the Bioproject PRJNA437375.

Ns, ambiguous nucleotides; N50, N20, and N80, sequence length of the shortest contig at 50, 20, and 80% of the total genome length.

^*a*^Sequences without genes and under 10 kbp have been removed.

The repeat content (Supplementary Table S3) was 51% of the total genome (656 355 643 bp, after excluding ambiguous bases, Ns), which is close to the 46% repeat content in foxtail millet (*S. italica*) (Zhang *et al.*, 2012). We found a large number of *Gypsy* and *Copia* LTRs, which represent 24% and 9.5% of the total genome excluding Ns. These transposons and proportions are also very similar to those observed in foxtail millet. We compared the divergence between the flanking tails in the LTRs (Supplementary Fig. S2) and identified a single very recent burst of LTR *Gypsy* activity around 0.6 million years ago (MYA; Kimura=0.042±0.026) and of LTR *Copia* also around 0.6 MYA (Kimura=0.041±0.027). Other repeat elements, including LINEs (long interspersed nuclear elements), simple repeat patterns of the sequence, satellites, and transposons were much less common, except for En/Spm DNA transposons observed in 4.2% of the genome.

We annotated 42 232 coding genes, which included 42 359 predictive ORFs, as well as 875 non-coding genes without a predicted ORF (Supplementary File 2 at Zenodo). Together these transcripts and non-coding genes define 43 234 targets for the expression analysis. A total of 35 982 of the coding transcripts had a homologous protein in the NCBI non-redundant (nr) database. In 58% of the cases, the top hit was an *S. italica* sequence (Supplementary Fig. S3). Among those 35 982, 33 963 were functionally annotated with at least one GO term, and 39 488 had an InterPro annotation. We also identified the best reciprocal hits with *A. thaliana*, rice, *Panicum halli* Vasey, foxtail millet, and *S. viridis* (L.) Beauv; and the top homologous protein in Uniprot (Supplementary File 3 at Zenodo). We aligned the transcripts and proteins from five sequenced species in the subfamily Panicoideae, foxtail millet, *S. viridis*, maize, *P. halli*, and switchgrass (*P. virgatum* L.) on the WGA, and found that on average 78% and 72% of the transcripts and proteins aligned with an identity >0.7, respectively (Supplementary Table S4).

### Comparative genomics with related grasses

We could assign 35 831 *Brachiaria* proteins to an eggNOG orthologous group (Powell *et al.*, 2014), and 13 570 proteins could be further annotated with GO terms from the eggNOG database. We also assigned the proteins from other species in the Poaceae family to these eggNOG orthologous groups for Poaceae (poaVIR) in the eggNOG database in order to identify shared clusters of proteins among these species (Supplementary Fig. S4; Supplementary File 4 at Zenodo). More than 70% of the clusters of proteins had double or triple the number of proteins in switchgrass than the other species because of relatively recent whole-genome duplication events (Supplementary Table S5). Approximately 20% of the clusters in maize and *B. ruziziensis* had two proteins. From this analysis, we also estimated that there are nearly 2000 proteins in other close species that were missed in our *Brachiaria* assembly.

We estimated the divergence between these species based on the Kimura divergence values between pairs of orthologous proteins in 6450 clusters of proteins that included only one member from each species (*P. virgatum* was excluded from this analysis; Supplementary Fig. S5). By assuming an average substitution rate of twice (diploid) 1.3×10^–8^ (Schmutz *et al.*, 2014), we estimated that *Brachiaria* diverged from the other Paniceae genera, *Setaria* and *Panicum*, ~13.4–15.5 MYA, while the split of the Paniceae and Andropogonodae tribes of subfamily Panicoideae took place ~23.8–26.3 MYA (Supplementary Fig. S6).

**Fig. 3.**
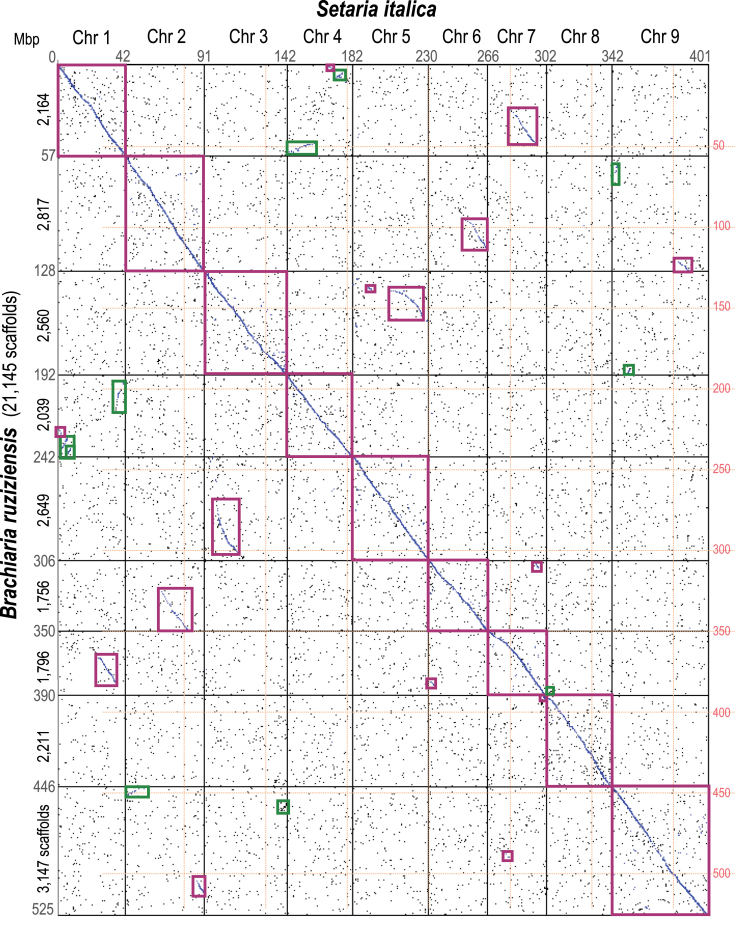
Synteny between the *Brachiaria ruziziensis* and foxtail millet (*Setaria italica*) genomes. The 36 synteny blocks >1 Mbp and translocations are highlighted in purple or green boxes according to their direction.

The 23 076 scaffolds in the WGS longer than 10 kbp or with at least one annotated gene (533.9 Mbp) were aligned with the nine chromosomes of foxtail millet, the closest relative with a high quality sequenced genome (Zhang *et al.*, 2012). Up to 21 145 of the 23 076 scaffolds (91.6 %), which comprise 525.1 Mbp, could be aligned (Fig. 3; Supplementary File 5 at Zenodo). Furthermore, we assigned chromosomal positions to 41 847 coding genes (41 974 transcripts) contained in these anchored sequences (Supplementary File 6 at Zenodo). We identified 59 synteny blocks, 36 of which were longer than 1 Mbp in both species (Fig. 3). There were three large translocations when comparing the foxtail millet and *B. ruziziensis* genomes between chromosomes 1 and 7, 2 and 6, as well as 3 and 5. Four inversions (smaller than the translocations) were identified between tails in chromosomes 1 and 4 (both ends), 2 and 9 (proximal end), and 2 and 3 (proximal end).

### QTL mapping in the interspecific *B. ruziziensis*×*B. decumbens* population

Between 78.8% and 91.5% of the GBS reads from each 169 interspecific progeny and the parents could be aligned in the assembly. After filtering, there was an average of 81 831 SNPs and 15 595 indel sites per sample. In total, 799 155 polymorphic sites were called in the population; 85.7% of these sites were SNPs. After filtering, 15 074 SNP sites were homozygous in the female parent (*B. ruziziensis* BRX 44-02) and heterozygous in the male parent (*B. decumbens* CIAT 606) (annotated as nnxnp markers in Joinmap), 4 891 sites were heterozygous in only the female parent (lmxll), and 1 652 were heterozygous in both parents (hkxhk). We classified 4 817 nnxnp and 1 252 lmxll sites as SDAs (or ‘simplex’ markers) based on their 1:1 heterozygous/homozygous segregation ratio in the progeny and used them in the genetic map construction. We used in our analysis the genetic map of the male parent (*B. decumbens* CIAT 606), which included 4 427 markers placed in 18 linkage groups (LGs; Supplementary Fig. S7; Supplementary File 7 at Zenodo). This corresponds to the number of base chromosomes expected in an allotetraploid (2*n*=4*x*=36) *Brachiaria* population. LGs had an average length of 74.7±22.7 cM. Using the same raw data, a joint genetic map of both parents and composed of 36 LGs was generated in Worthington *et al.* (2016). Two LGs were assigned to each of the nine base chromosomes of *B. ruziziensis* based on alignments of each SNP to the WGA and named following the numbering system used for foxtail millet. We also aligned the WGA scaffolds with the genetic map to compare the collinearity between the position of each marker in the genetic map and genome assembly (Supplementary File 7 at Zenodo.

QTL mapping was performed to identify genetic regions associated with relative root length, biomass, and root diameter under control and Al^3+^ stress conditions in the interspecific mapping population, and the ratio between stress and control (stress/control). Three hundred and seventy-one markers had a LOD >3 associated with one or more of the traits. Two hundred and twelve WGS scaffolds contained at least one marker with a LOD >3 (Supplementary File 7 at Zenodo).

**Fig. 4.**
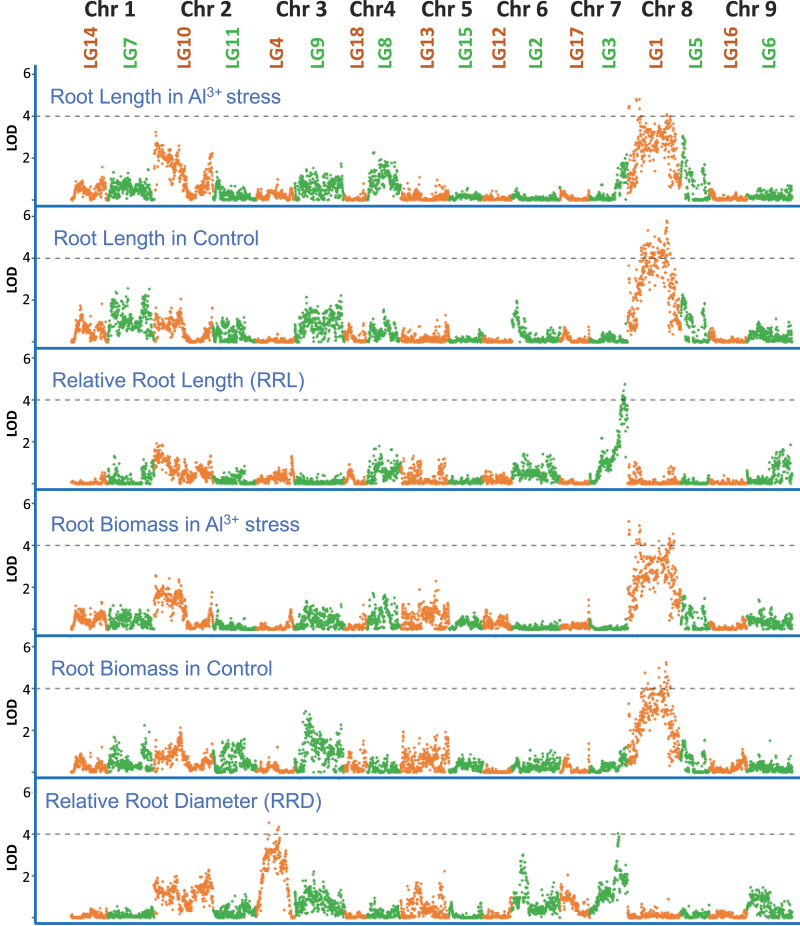
A total of 4427 genetic markers placed in 18 linkage groups. We defined three QTLs each in LG1 (Chr 8) for root length and biomass under control and Al^3+^ stress conditions, LG3 (Chr 7) for relative root length ratio and relative average root diameter ratio, and LG4 (Chr 3) for average root diameterratio.

Three significant QTLs were identified (Fig. 4). The first QTL, which peaked at 5.22, 12.65, 25.797, and 26.027 cM and extended from 5.22 cM to 32.481 cM on LG1 (Chr 8), was associated with root length and biomass under both control and Al^3+^ stress conditions. The second QTL, which peaked at 79.738 cM and 96.802 cM and extended from 79.738 cM to 98.851 cM on LG3 (Chr 7) was associated with RRL and root diameter under stress/control conditions. The last QTL extended from 49.12 cM to 62.127 cM (peak at 50.423 cM) on LG4 (Chr 3) and was only associated with RRD under stress/control conditions. These three QTLs each explained from 12.8% to 16.1% of the phenotypic variance for the associated traits (Supplementary Table S6).

### Transcriptional differences during stress between *Brachiaria* species

**Fig. 5.**
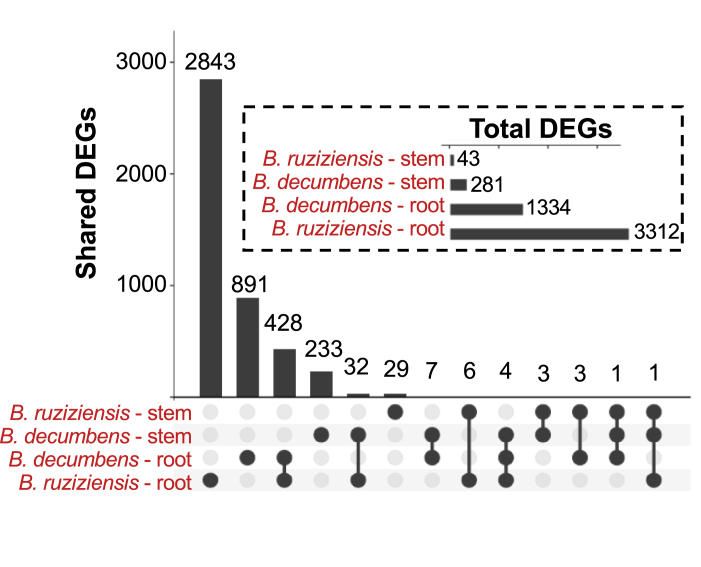
Differentially expressed genes (DEGs) in roots and stems from the susceptible genotype *B. ruziziensis* BRX 44-02 and the tolerant genotype *B. decumbens* CIAT 606 (cv. Basilisk) between control and high (200 μM AlCl_3_) aluminium cation concentrations in hydroponic experiments at CIAT.

We performed RNA-seq from the stem and root tissue samples extracted from *B. decumbens* CIAT 606 and *B. ruziziensis* BRX 44-02 after growing for 3 d in control (0 μM AlCl_3_) or high (200 μM AlCl_3_) aluminium cation concentration hydroponic solutions. When the normalized counts for all the genes were used to cluster the samples, these clusters firstly grouped by tissue, secondly by genotype, and thirdly by treatment (Supplementary Fig. S8). There were 4481 DEGs in total, with 3996 of these differentially regulated in a single genotype and tissue (Fig. 5). Among these 4481 DEGs, 4162 genes were differentially expressed in roots only, 265 in stems only, and 54 in both tissues.

The three QTL regions contained 918 genes; 581 in LG1, 153 in LG3, and 184 in LG4. Of these, 84 were DEGs (Supplementary File 8 at Zenodo). Twenty-seven DEGs were annotated as membrane components, 21 were annotated as involved in response to hormones or biotic/abiotic stress, and 23 were annotated as binding to different molecular compounds, including ATP/ADP/GTP, metal ions, and DNA. A total of 34 of the 87 genes were not annotated with one of these previous GO terms, but several were annotated with several GO terms.

**Fig. 6.**
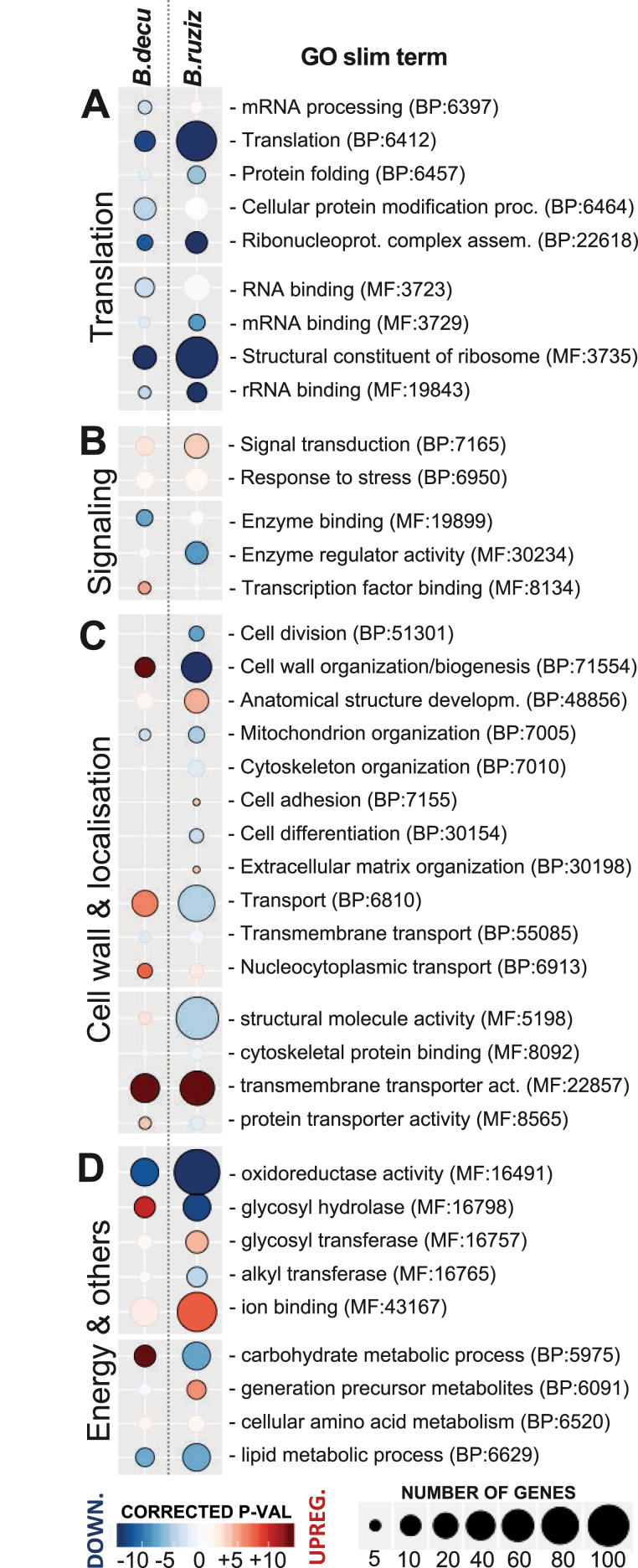
Enrichment analysis of the GO slim terms over-represented among differentially expressed genes that are either up-regulated (red) or down-regulated (blue) during stress in roots from the susceptible *B. ruziziensis* BRX 44-02 (*B.ruziz*) or the tolerant genotype *B. decumbens* CIAT 606 cv. Basilisk (*B.decu*) genotypes.

Enrichment analysis of GO terms over-represented among DEGs in each species allowed us to identify the biological processes (BP) and molecular functions (MF) that are similarly or differently regulated among them (Supplementary Files 9 and 10 at Zenodo). After annotating the genes with the full set of GO terms (Supplementary Figs S9, S10), we also simplified the results to ‘GO slim’ terms, containing the subset of higher level terms from the GO resource (Fig. 6; Supplementary Table S7). For the enrichment analysis, we considered either up-regulated or down-regulated genes in each sample. There was limited overlap among GO terms based on the DEGs included in each term (Supplementary Fig. S11).

Five MF terms related to RNA/mRNA/rRNA binding with the ribosome (MF:3723, 3729, 19843, 3735, and 5198) and two BP terms, ‘translation’ and ‘ribonucleoprotein complex assembly’ (BP:6412 and 22618), were significantly enriched among down-regulated genes during stress in *B. ruziziensis* (146 genes) and *B. decumbens* (39 genes).

Genes annotated as ‘transmembrane transporters’ (MF:22857) were highly over-represented among DEGs up-regulated during stress (44 in *B. decumbens* and 70 in *B. ruziziensis*). Fifteen differentially expressed transmembrane transporters were common to both species, and five were annotated as induced by aluminium and are discussed later.

Several GO terms showed contrasting regulation between both species. For example, ‘Cell wall organization and biogenesis’ was enriched in both species and included 51 down-regulated genes in *B. ruziziensis*, but 17 up-regulated genes in *B. decumbens*. Most of these genes were peroxidases and expansin proteins induced by various plant hormones (e.g. ethylene, gibberellin, and auxin) and involved in toxin removal during oxidative stress (Kochian *et al.*, 2015). The GO term ‘glycosyl hydrolase’ was also enriched among down-regulated genes in *B. ruziziensis* (39 genes), but among up-regulated genes in *B. decumbens* (18 genes). Most of the genes annotated as ‘glycosyl hydrolases’ were also annotated as involved in ‘carbohydrate metabolism’ (BP:5975).

Several of the DEGs that exhibited high fold changes in both species were involved in phospholipid, phosphate, magnesium, auxin, nitrate, and miRNA transport in the cell (genes 6944G2, 12087G2, 8068G4, 26018G2, 4534G2, and 2708G4; Table 2).

**Table 2.** *Brachiaria* genes differentially expressed during Al^3+^ treatment in *Brachiaria* highlighted in our analysis

GeneID	Bruz	Bdec	Gene name	*P. halli*	*S. italica*	*S. viridis*	*A. thaliana*	Uniprot	Function and induction
29G2	0.71	0.17	STOP1	3G044400	7G287100	7G299300	AT1G34370	STOP1_ORYSJ	Zinc-finger transcription factor involved in Al tolerance
61G2	0.88	0.23	Citrate transporter detoxification 42	9G552400	9G489200	9G493200	AT1G51340	DTX42_ARATH	Citrate transporter responsible for citrate exudation into the rhizosphere to protect roots from Al toxicity. Up-regulated by Al. Expressed in roots, but not in shoots.
94G8	7.68	2.36	P transmembrane transporter	7G133200	7G082600	7G090100	AT4G13420	HAK1_ORYSJ	In the QTL. Potassium transmembrane transported induced by P starvation exclusively in roots.
126G26	1.83	0.95	STOP1	6G306100	6G252000	6G256600	AT5G22890	STOP1_ORYSJ	Transcription factor involved in Al tolerance
259G14	2.36	1.43	Citrate transporter protein detox. 42	9G201000	9G204700	9G203100	AT1G51340	DTX42_ARATH	Citrate transporter responsible for citrate exudation into the rhizosphere to protect roots from Al toxicity. Up-regulated by Al. Expressed in roots, but not in shoots.
462G24	–2.35	0.38	AMLT10	4G147500	4G162600	4G130000	–	K3XVN8_SETIT	Al-activated malate transporter 10-like
598G8	–0.97	–2.58	Cytochrome P450	7G003400	1G019400	1G019300	AT5G07990	C93G1_ORYS	In the QTL. Metal-binding transmembrane P450 cytochromes
675G12	4.27	3.89	Citrate transporter protein detox. 42	5G042400	9G489200	9G493200	AT1G51340	DTX42_ARATH	Citrate transporter responsible for citrate exudation into the rhizosphere to protect roots from Al toxicity. Up-regulated by Al. Expressed in roots, but not in shoots.
869G16	1.36	1.83	Cysteine XCP1 protease	2G435100	8G204200	8G213900	AT4G35350	XCP1_ARATH	In the QTL. A proteinase that may have a developmental senescence-specific cell death function during apoptosis and metal detoxification
1615G2	2.69	1.29	STOP1	6G306100	6G252000	6G256600	AT5G22890	STOP1_ORYSJ	Zinc-finger transcription factor involved in Al tolerance
1984G2	1.09	0.82	SLK2	4G345700	4G015400	4G015300	AT5G62090	SLK2_ARATH	DNA-binding adaptor subunit of the SEU–SLK2 transcriptional co-repressor of abiotic stress (e.g. salt and osmotic stress) response genes by facilitating auxin response and sustaining meristematic potential.
2499G8	1.18	–0.15	OsTITANIA/AtOBE3	9G552000	9G488800	9G492800	AT1G14740	TTA1_ORYSJ	Widely expressed transcription factor that functions as regulator of metal transporter genes responsible for delivery of essential metals to shoots and normal plant growth. Required for the maintenance of metal transporter gene expression, such as IRT1, IRT2, ZIP1, ZIP9, NRAMP1, and NRAMP5.
2708G4	1.64	1.94	Auxin transport protein BIG	2G178200	2G154800	2G160200	AT3G02260	BIG_ORYSJ	Auxin-mediated developmental responses (e.g. cell elongation, apical dominance, lateral root production, inflorescence architecture, general growth, and development).
3096G6	0.51	0.27	ALS1	9G083700	9G088000	9G086400	AT5G39040	AB25B_ORYSJ	Metal transporter involved in the sequestration of Al into vacuoles, which is required for cellular detoxification of Al. Induced by Al in roots. Expressed in primary roots and lateral roots.
3672G6	2.54	2.26	NRAT1	1G025100	1G098100	1G097200	AT1G80830	NRAT1_ORYSJ	Metal transporter of Al^3+^, but not divalent cations Fe^2+^, Mg^2+^, Cd^2+^. Involved in Al tolerance by taking up Al in root cells, where it is detoxified by chelation with organic acid anions and sequestration into the vacuoles. Induced by Al in roots. Positively regulated by ART1.
4534G2	4.74	0.43	SAD2 importin beta-like transporter	7G101600	7G051600	7G057300	AT2G31660	SAD2_ARATH	Involved in the regulation of the abscisic acid (ABA)-mediated pathway in response to cold or salt stress, UV-B responses. Involved in trichome initiation. Negative regulator of miRNA activity. Expressed in roots, epidermal and guard cells of leaves, stems. and siliques
5233G2	1.07	1.18	XTH23	4G029300	4G246200	4G258800	AT4G25810	XTH23_ARATH	Xyloglucan endotransglucosylase hydrolase protein 23 cleaves and religates xyloglucan polymers, an essential constituent of the primary cell wall, and thereby participates in cell wall construction of growing tissues. Up-regulated by ABA.
5400G2	–1.38	–0.05	XTH	8G197800	8G142000	8G151900	AT5G13870	XTH5_ARATH	Cleaves and religates xyloglucan polymers, an essential constituent of the primary cell wall, and thereby participates in cell wall construction of growing tissues. Strongly down-regulated by ABA. Root specific.
6544G2	–0.07	0.95	XTH32	2G372300	2G313800	2G324800	AT2G36870	XTH8_ORYSJ	Xyloglucan endotransglucosylase hydrolase protein 32 cleaves and religates xyloglucan polymers, an essential constituent of the primary cell wall, and thereby participates in cell wall construction of growing tissues. May be involved in cell elongation processes. Induced by gibberellic acid (GA_3_).
6944G2	1.92	2.01	Phospholipid-transporting ATPase	4G247400	4G147300	4G165700	AT1G26130	ALA12_ARATH	Involved in transport of phospholipids.
7361G2	2.26	1.39	STAR2/ALS3	3G099200	3G043600	3G044400	AT2G37330	STAR2_ORYSJ	Associates with STAR2 to form a functional transmembrane ABC transporter required for detoxification of Al in roots. Can specifically transport UDP-glucose. Induced by Al in roots. Positively regulated by ART1. Root specific.
8068G4	2.77	2.12	Mg transporter MRS2-D	7G150800	7G099600	7G107500	AT3G19640	MRS2D_ORYSJ	Putative magnesium transporter.
8234G4	–1.40	–1.30	Uncharacterized	–	–	–	–	A0A3L6T2E7_PANMI	In the QTL. Uncharacterized protein.
8440G2	0.88	–1.68	Cytochrome P450	6G227500	2G094000	2G096800	AT4G36380	C90C1_ARATH	In the QTL. Metal-binding transmembrane P450 cytochromes
8448G4	0.59	1.26	STAR1	4G029500	4G246800	4G259400	AT1G67940	STAR1_ORYSJ	Associates with STAR2 to form a functional transmembrane ABC transporter required for detoxification of Al in roots. Can specifically transport UDP-glucose. Induced by Al in roots. Positively regulated by ART1. Root specific.
11634G4	0.04	4.75	AMLT1	7G143300	7G007400	7G102000	AT3G11680	ALMT8_ARATH	Malate transporter.
11683G4	1.00	0.66	XTH27	–	–	–	–	A0A1D6K9K3_ZM	Xyloglucan endotransglucosylase hydrolase protein 27 cleaves and religates xyloglucan polymers, an essential constituent of the primary cell wall, and thereby participates in cell wall construction of growing tissues.
12087G2	1.31	3.21	Phosphate transporter pho1-2	1G440700	1G360400	1G367100	AT3G23430	PHO12_ORYSJ	Involved in the transfer of inorganic phosphate (Pi) from roots to shoots. Not induced by Pi deficiency in roots. Specifically expressed in roots.
26018G2	1.08	0.98	NRT1 nitrate transporter	9G390400	9G327900	9G333900	AT2G26690	PTR27_ARATH	Low-affinity proton-dependent nitrate transporter. Up-regulated in the shoots by nitrate, but no changes in the roots.
30107G2	3.24	3.02	NRAT1	2G074500	1G098100	1G097200	AT1G80830	NRAT1_ORYSJ	Metal transporter of Al^3+^, but not divalent cations Fe^2+^, Mg^2+^, Cd^2+^. Involved in Al tolerance by taking up Al in root cells, where it is detoxified by chelation with organic acid anions and sequestration into the vacuoles. Induced by Al in roots. Positively regulated by ART1.

GeneID, gene number in our reference genome; Bruz/Bdec, differential expression fold change values in *B. ruziziensis* and *B. decumbens* under Al^3+^ stress; significant *P*-values <0.05 are coloured. *P. halli/S. itallica/S. viridis/A. thaliana*, top Blastp result in each of these species; Uniprot and ‘Function and induction’, top Blastp result and associated annotation in the Uniprot database.

By reanalysing the data from Salgado *et al.* (2017), we could also compare the enriched GO terms between *B. decumbens* cv. Basilisk exposed to 200 μM AlCl_3_ for 8 h and 72 h (Supplementary Fig. S12; Supplementary Table S7). This comparison evidenced differences between 8 h and 72 h in the same genotype in GO terms related to RNA translation and response to stress (BP:6950), among others.

## Discussion

### Forward genetics and newly produced genomic resources to identify candidate loci for abiotic stress tolerance

The adverse effects of soil acidity on crop production are associated with different mineral toxicities and deficiencies. Aluminium cation toxicity has long been established to be the single most important limiting factor of acidic soil productivity (Eswaran *et al.*, 1997). All *Brachiaria* species show some tolerance to Al^3+^ toxicity, particularly compared with other grasses such as wheat, rice, and maize (Arroyave *et al.*, 2013; Kochian *et al.*, 2015). While most crops reduce root growth to 50% when exposed to 5 μM Al^3+^, *Brachiaria* species need to be exposed to up to 35 μM to exhibit a reduced root growth of 25% (Poschenrieder *et al.*, 2008).

Natural adaptation to acid soils can be measured by estimating root vigour in low pH conditions and tolerance to high concentrations of Al^3+^ (Wenzl *et al.*, 2006). In our experiment, *B. decumbens* CIAT 606 (cv. Basilisk) root morphology (length, biomass, and root diameter) was less affected than that of *B. ruziziensis* BRX 44-02 after growing for 20 d in control and high 200 μM Al^3+^ concentration hydroponic solutions (Fig. 1). Under Al^3+^ toxic conditions, root tips are damaged (so limiting root elongation), lateral roots are inhibited, and root hairs are deformed (Rao *et al.*, 2016). In a 21 d long-term study using a similar experimental protocol, four *B. decumbens* and four *B. ruziziensis* genotypes showed intermediate tolerance of Al^3+^, one *B. ruzizensis* genotype was sensitive, and only the *B. decumbens* cultivar Basilisk was unaffected by high Al^3+^ (Bitencourt *et al.*, 2011). Interestingly, Furlan *et al.* (2018) recently reported a higher Al^3+^ tolerance in *B. brizantha* cv. Xaraes than in *B. decumbens* cv. Basilisk.

The three QTLs we identified were of moderate effect (LOD scores <6, each accounting 12.8–16.1% of observed phenotypic variance), but were observed for several root traits (Fig. 4). DEGs in the QTLs highlighted a link with membrane transport (including metal ions), regulation and signalling (binding to DNA), and energy (carbohydrate metabolism and binding to ATP/ADP/GTP) molecular mechanisms in the three QTLs. The three QTLs were associated with similar molecular functions. Eight genes in the QTLs were differentially expressed in both species, including two metal-binding transmembrane P450 cytochromes (8440G2 and 598G8), one root-exclusive potassium ion membrane transporter (94G8) induced by potassium starvation (Banuelos *et al*., 2002), a cysteine XCP1 protease (869G16) that may have a developmental senescence-specific cell death function during apoptosis and heavy metal detoxification (De Michele *et al.*, 2009), and a protein (8234G4) not found in other related species (Table 2).

We are also making publicly available the genome assembly and gene annotation of a diploid accession of *B. ruziziensis* (accessible at NCBI with accession no. GCA_003016355). This diploid accession collected in Burundi is probably the genotype that was mutagenized with colchicine to produce CIAT BRX 44-02, the autotetraploid *B. ruziziensis* progenitor of the interspecific population analysed in this study. Our transcriptomic study and comparative genomics analysis are examples of its utility. While tetraploid *Brachiaria* accessions are the main accessions used for breeding, we opted for a non-polyploid diploid accession because *Brachiaria* grasses are heterozygous outcrossing species.

The assembly was partially scaffolded to the pseudomolecule level using a genetic linkage map of *B. decumbens* CIAT 606 containing ~5000 markers (Supplementary Fig. S7). This genetic map had previously been assembled without a genome (Worthington *et al.*, 2016), but a higher number of markers were incorporated by conducting SNP calling using this new reference. The incorporation of long reads could improve this assembly and allow for polyploid *Brachiaria* genome references in the near future. Because of the limited number of markers, almost all the scaffolds were also placed on the high-quality foxtail millet genome. While we identified three large chromosomal translocations (Fig. 3), there was very high collinearity between the two species as previously observed (Worthington *et al.*, 2016) and as might be predicted from species that we quantified as diverged ‘only’ 13–15 MYA.

### Differentially expressed genes associated with internal tolerance mechanisms to Al^3+^ in *Brachiaria*

Aluminium internal tolerance mechanisms involve either modification of the properties of the root cell wall, or the uptake and sequestration of Al^3+^ once it enters the plant (Kochian *et al.*, 2015). Ramos *et al.* (2012) observed that *B. decumbens* accumulated Al^3+^ in the mucilage layer of root apices, which reduced the quantity of Al^3+^ reaching the cell wall and crossing the plasma membrane. Arroyave *et al.* (2013) suggested that the presence of a complex exodermis in *B. decumbens*, absent in *B. ruziziensis*, may contribute to a more efficient exclusion of Al^3+^. On the other hand, a higher concentration of root pectin measured in *B. ruziziensis* during stress could be evidence of increased apoplastic aluminium binding (Horst *et al.*, 2010).

Changes in the structural properties of cell wall carbohydrates are mediated by expansins, endo-β-1,4-glucanases, xyloglucan transferases and hydrolases (XTH) (e.g. AtXTH31 and AtXTH15), and pectin methylesterases (Yang *et al.*, 2011; Kochian *et al.*, 2015). Contrasting regulation (up-regulated in one species but down-regulated in the other) in enriched GO terms between *B. decumbens* and *B. ruziziensis* (Fig. 6; Supplementary Figs S9, S10) suggests different regulation of genes associated with xyloglucan transfer (GO:16762), glycosyl hydrolase (MF:16798), and oxidation (GO:52716). This contrasting enrichment was mostly due to 12 XTH proteins that were down-regulated during stress in *B. ruziziensis*, but not in *B. decumbens*. One of them (5400G2) was located in the QTL region on LG1 (Chr 8; Table 2). There were three additional differentially expressed XTH proteins that were up-regulated during stress: 5233G2 (AtXTH23) only in *B. decumbens*, and 6544G2 (AtXTH32) and 11683G4 (AtXTH27) in both *B. decumbens* and *B. ruziziensis* (Table 2). Related to this, AtSLK2 is involved in cell wall pectin methylesterification in response to Al^3+^ stress (Geng *et al.*, 2017). The *Brachiaria* homologous gene to SLK2 (1984G2) was up-regulated in both *B. ruziziensis* and *B. decumbens*, but only differentially expressed under stress in *B. decumbens* (Table 2).

Once Al^3+^ has entered the root, the uptake and sequestration of Al^3+^ includes molecular binding and eventually compartmentation of the toxin. ALS (aluminium-sensitive) transporters and NRAM metal ion transporters have been proposed as the critical proteins in Al^3+^ localization to the tonoplast and other cell organelles, and away from the sensitive root tips in *A. thaliana* and rice (Larsen *et al.*, 2007; Huang *et al.*, 2010, 2012). Specifically, an NRAM aluminium transporter (NRAT1) localized in the plasma membrane appears to be expressly involved in storing Al^3+^ in root vacuoles in rice and maize. In *Brachiaria*, we identified 21 NRAM proteins, but only three of them were differentially expressed (Table 2): the homologous gene (2499G8) to OsTITANIA (AtOBE3/ATT1), which is the transcription factor that functions as a regulator of NRAM and other metal transporter genes (Tanaka *et al.*, 2018), and two of the three *Brachiaria* proteins showing close homology to NRAT1 (3672G6 and 30107G2; Table 2). These were significantly up-regulated in both *B. decumbens* and *B. ruziziensis* during stress. Eight genes in the *Brachiaria* genome are homologous to ALS1 and annotated as aluminium-induced ABC transporters (Supplementary File 3 at Zenodo), but only one gene (3096G6) was up-regulated during stress in *B. ruziziensis* (Table 2).

In rice, the complex formed by STAR1 and STAR2/AtALS3 (sensitive to aluminium rhizotoxicity) is involved in aluminium-induced alterations of the cell wall composition related to less aluminium binding in the apoplast (Huang *et al.*, 2009, 2010). These ABC transporters appear to mediate the efflux of UDP-glucose into the cell wall, which could alter the cell wall composition and lead to a reduction in aluminium binding capacity (Kochian *et al.*, 2015). Fifteen proteins were homologous to STAR/ALS in *Brachiaria*, but only two were differentially expressed: gene 8448G4 homologous to OsSTAR1, and gene 7361G2 homologous to OsSTAR2 (Table 2). STAR1 and STAR2 were up-regulated in both *B. ruziziensis* and *B. decumbens* during stress.

### Differentially expressed genes associated with external tolerance mechanisms to Al^3+^ in *Brachiaria*

Most aluminium-tolerant crops additionally rely on external restriction to prevent the uptake of aluminium and its entry into the root cells through the release of anionic organic acids in the rhizosphere that chelate the Al^3+^ (Kochian *et al.*, 2015; Rao *et al.*, 2016). However, *Brachiaria* appears not to rely on secreted organic acids since we observed no difference in secreted organic acids between *Brachiaria* species (Fig. 6; Supplementary Figs S9, S10), as previously reported (Wenzl *et al.*, 2001; Arroyave *et al.*, 2013). Similarly, internal detoxification primarily occurs in the sensitive rice variety Modan, while external mechanisms are observed in tolerant varieties (Roselló *et al.*, 2015). The lack of correlation between exudation and resistance has also been observed in rice (Ma *et al.*, 2002; Famoso *et al.*, 2010). Furthermore, tolerant *B. decumbens* accessions secreted 3–30 times less organic acids than sensitive species such as maize and wheat (Wenzl *et al.*, 2001; Arroyave *et al.*, 2018). However, while *B. decumbens* citrate exudation was ~200 times lower than that observed in aluminium-tolerant rice, the same study evidenced high oxalate exudation in *B. decumbens* roots, but only between 24 h and 36 h after exposure to the toxic concentration (Arroyave *et al.*, 2018). It appears that other mechanisms of resistance overshadow the impact of root exudation.

Two families of membrane transporters, aluminium-activated malate transporter (ALMT) and the multidrug and toxic compound extrusion (MATE) family, are responsible for plasma membrane malate and citrate efflux, respectively (Raman *et al.*, 2005; Guimaraes *et al.*, 2014; Rao *et al.*, 2016). Citrate is a much stronger chelating agent for Al^3+^ than malate (Ma, 2000). In *Brachiaria*, we identified three citrate MATE proteins, which were homologous to FRDL (ferric reductase defective like) proteins and up-regulated with large fold changes in *B. decumbens* only (61G2), or in both species (259G14 and 675G12) (Table 2). In rice, FRDL4 is responsible for aluminium-induced citrate efflux required for external detoxification (Yokosho *et al.*, 2011).

We identified 13 ALMTs in the genome. However, only two were differentially expressed: 11634G4 was up-regulated with a 4.74-fold change during stress in *B. decumbens*, and 462G24 was down-regulated with a 2.35-fold change in *B. ruziziensis* (Table 2). We identified a cluster with three contiguous non-differentially expressed ALMTs (5136G2, 5136G4, and 5136G6) in scaffold 5136 [LG3 (Chr. 7): 71.83–72.95 cM] ~10 cM from the QTL. This is consistent with the observation that copy number variation of ALMT correlated with aluminium resistance in rye and maize (Collins *et al.*, 2008; Maron *et al.*, 2013).

C_2_H_2_-type zinc-finger transcription factors STOP1 (ART1 in rice) and STOP2 regulate aluminium-induced expression of several MATE and ALMT genes in Arabidopsis and rice (Iuchi *et al.*, 2007; Yamaji *et al.*, 2009; Kobayashi *et al.*, 2014). We identified three transcription factors homologous to AtSTOP1, one differentially expressed in *B. ruziziensis* (29G2) and another two (3833G12 and 243G34) which were not (Table 2). We also identified two transcription factors homologous to AtSTOP2, both differentially expressed with high fold change in *B. ruziziensis* and *B. decumbens* (126G26 and 1615G2) (Table 2). All five were homologous to OsART1, the STOP1 homologue in rice, which up-regulated at least 31 genes in an aluminium-dependent manner, including STAR1, FRDL, and NRAMP proteins (Yamaji *et al.*, 2009; Kochian *et al.*, 2015).

The ‘transmembrane transporters’ GO term (MF:22857) was highly over-represented (Fig. 6) among up-regulated DEGs during stress in both species (44 genes in *B. decumbens* and 70 genes in *B. ruziziensis*). Fifteen transmembrane transporters were differentially expressed in both species, and five were annotated as induced by aluminium: NRAT1 (3672G6), STAR1 (8448G4), ALS3 (7361G2), and two citrate MATE transporters (675G12 and 259G14). All these have been highlighted in Table 2, up-regulated with high fold changes in both species, and are probably indispensable genes for aluminium tolerance in *Brachiaria*.

The present work relied on simple phenotypic root traits (root length, average root diameter, and root biomass) that have been broadly used in screening for genotypic differences in Al^+3^ tolerance among *Brachiaria* (Wenzl *et al.*, 2006) by us and others (Arroyave *et al.*, 2011; Salgado *et al.*, 2017). Furthermore, our response screening was limited to a time point at 3 d after the imposition of stress for the RNA-seq experiment (Supplementary Fig. S8) or 20 d for the QTL mapping experiment. As a result, essential traits and processes were irrefutably missed and thereby absent in any analysis following a similar scheme.

Since root toxic species and limited nutrient availability occur together in acidic soils and are not homogeneously distributed, plant roots have to deal with different levels of these factors during their growth by regulating the available genetic repertoire throughout the growing season (Liao *et al.*, 2006; Liang *et al.*, 2013). The over-representation of uncharacterized genes associated with ‘RNA translation’ and ‘response signalling’ (Fig. 6) shows that critical information is unknown. The increasing accessibility of high-throughput phenomics will allow the capture of highly informative plant traits (e.g. growth and senescence) to enable dissection of the genetic components of Al^3+^ tolerance at different stages (e.g. acclimation, quiescence, or recovery periods) with unprecedented detail.

In this work, we have presented a comprehensive analysis of the molecular mechanism linked to aluminium tolerance in *Brachiaria* species. By assembling and annotating a diploid genotype of *B. ruziziensis*, we have developed the capability for genomic-based studies of desirable phenotypic traits. Using this resource, we have identified three QTLs associated with root morphology during Al^3+^ stress in a hybrid population from high and low tolerance accessions. We have also identified several genes and molecular responses that impact on different aspects of signalling, cell wall composition, and active transport as a response to aluminium stress. *Brachiaria* tolerance appears to rely upon the same genes as those in rice. However, we found that external mechanisms such as chelation of Al^3+^, common in other grasses, might not be very important in *Brachiaria.* Numerous genes involved in RNA translation showed contrasting regulation between 8 h and 72 h of Al^3+^ stress (Supplementary Fig. S12); that is, the functions up-regulated at early stages were down-regulated at later stages, or vice versa. This contrasting regulation of RNA translation and response signalling suggests response phasing is critical to high Al^3+^-tolerant *Brachiaria* species. The newly annotated draft genome represents an essential base upon which other aspects of *Brachiaria* biology can be studied.

## Supplementary data

The following supplementary data are available at *JXB* online.

Table S1. Root length, diameter, and biomass in the *B. decumbens* CIAT 606 and *B. ruziziensis* BRX 44-02 (cv. Basilisk) progenitors after growing for 20 d in control and high 200 μM AlCl_3_ concentration hydroponic solutions.

Table S2. Statistics of the intermediate steps, alternative assemblies, final assembly, and pseudo-molecules for the *B. ruziziensis* CIAT 26162 genome.

Table S3. Classification of the repeat content in the *Brachiaria* genome.

Table S4. Alignment of the transcripts and proteins from five sequenced species in the Panicoideae subfamily in the *Brachiaria ruziziensis* genome.

Table S5. EggNOG clusters in six sequenced species in the Panicoideae subfamily classified by number of proteins per cluster.

Table S6. Peak and interval positions for the identified QTLs, as well as the corresponding *S. italica* chromosome.

Table S7. Enrichment analysis of the GO SLIM terms over-represented among differentially expressed genes in *B. ruziziensis* BRX 44-02 (Bruz), *B. decumbens* CIAT 606 (cv. Basilisk) (Bdec), or PRJNA314352 from Salgado *et al.* (2017).

Fig. S1. 31mer frequency analysis comparing the short read assemblies produced with *Platanus assembler* or ABySS and SOAP2.

Fig. S2. Divergence (Kimura) rates between the flanking tails in each *Gypsy* and *Copia* LTR duplication event in the *Brachiaria* genome.

Fig. S3. Species of the top Blastp hit for each of the 35 982 coding transcripts which had a homologous protein in the NCBI non-redundant (nr) database.

Fig. S4. Shared eggnog clusters of proteins among *Brachiaria ruziziensis* (Bruz), foxtail millet, *S. viridis*, maize, *Panicum halli*, and switchgrass.

Fig. S5. Kimura rates between homologous gene pairs between *B. ruziziensis* and sequenced relatives including foxtail millet, *S. viridis*, maize, and *P. halli.*

Fig. S6. Phylogenetic tree based on the nucleotide divergence rate between sequences in the same eggnog cluster from *B. ruziziensis* and sequenced relatives.

Fig. S7. The final genetic map for the *B. decumbens* CIAT 606 (cv. Basilisk) progenitor of the interspecific population included 4427 markers placed at LOD 10 in 18 linkage groups.

Fig. S8: RNA-seq from stem and root tissue samples extracted from the *B. decumbens* and *B. ruziziensis* progenitors. We also incorporated a reanalysis of public RNA-seq data (PRJNA314352) from *B. decumbens* var. Basilisks roots.

Fig. S9. Enrichment analysis of the ‘molecular function’ GO terms over-represented among differentially expressed up-regulated (red) or down-regulated (blue) genes in roots in *B. decumbens* CIAT 606 and *B. ruziziensis* BRX 44-02.

Fig. S10. Enrichment analysis of the ‘biological process’ GO terms over-represented among differentially expressed up-regulated (red) or down-regulated (blue) genes in roots in *B. decumbens* CIAT 606 and *B. ruziziensis* BRX 44-02.

Fig. S11. Correlation matrix plot among GO terms based on the differentially expressed genes included in each annotation.

Fig. S12. Comparison of the enriched GO Slim terms between *B. decumbens* cv. Basilisk exposed to 200 μM AlCl_3_ for 72 h and 8 h, the latter from the reanalysis of public raw data from Salgado *et al.* (2017).

eraa469_suppl_Supplementary_Materials

## Data Availability

Raw reads are deposited in SRA under accession PRJNA437375. The genome assembly is deposited at the NCBI with accession no. GCA_003016355 (https://www.ncbi.nlm.nih.gov/assembly/1650221). Individual scaffolds can be accessed at NCBI’s GenBank accession nos PVZT01000001–PVZT01102577. Additional datasets S1–S10, which contain the genome assembly, chromosomal anchoring in AGP format, gene models, position and function annotations, and enrichment analysis of Gene Ontology terms can be downloaded as individual files from Zenodo (http://dx.doi.org/10.5281/zenodo.3941963.
