# Supplementary material for: A new genome allows the identification of genes associated with natural variation in aluminium tolerance in Brachiaria grasses
Source: J Exp Bot. 2020 Oct 16;72(2):302–19. doi: 10.1093/jxb/eraa469 (PMC7853602; doi:10.1093/jxb/eraa469)

A new genome allows the identification of genes associated with natural variation in aluminium tolerance in *Brachiaria* grasses

- **Supplementary table S1:** Root length, diameter and biomass in the *B. decumbens* CIAT 606 and *B. ruziziensis* BRX 44-02 (cv. Basilisk) progenitors after growing for 20 days in control and high 200 μM $AlCl_3$ concentration hydroponic solutions.
- **Supplementary Table S2:** Statistics of the intermediate steps, alternative assemblies, final assembly and pseudo-molecules for the *B. ruziziensis* CIAT 26162 genome.
- **Supplementary Table S3:** Classification of the repeat content in the Brachiaria genome.
- **Supplementary Table S4:** Alignment of the transcripts and proteins from five sequenced species in the Panicoideae subfamily in the *Brachiaria ruziziensis* genome.
- **Supplementary Table S5:** EggNOG clusters in six sequenced species in the Panicoideae subfamily classified by number of proteins per cluster.
- **Supplementary Table S6:** Peak and interval positions for the identified QTLs, as well as corresponding *S. italica* chromosome.
- **Supplementary Table S7:** Enrichment analysis of the GO SLIM terms over-represented among DE genes in B. ruziziensis BRX 44-02 (Bruz), B. decumbens CIAT 606 (cv. Basilisk) (Bdec), or PRJNA314352 from Salgado *et al*. (2017).
- **Supplementary Figure S1:** 31mer frequency analysis comparing the short-reads assemblies produced with *Platanus assembler* or ABySS and SOAP2.
- **Supplementary Figure S2:** Divergence (Kimura) rates between the flanking tails in each *Gypsy* and *Copia* LTR duplication events in the *Brachiaria* genome.
- **Supplementary Figure S3:** Species of the top Blastp hit for each 35,982 of the coding transcripts which had a homologous protein in the NCBI non-redundant (nr) database.
- **Supplementary Figure S4:** Shared eggnog clusters of proteins among *Brachiaria ruziziensis* (Bruz), foxtail millet, *S. viridis*, maize, *Panicum halli* and switchgrass.
- **Supplementary Figure S5:** Kimura rates between homologous gene pairs between *B. ruziziensis* and sequenced relatives including foxtail millet, *S. viridis*, maize, and *P. halli*.

- **Supplementary Figure S6:** Phylogenetic tree based on nucleotide divergence rate between sequences in the same eggnog cluster from *B. ruziziensis* and sequenced relatives.
- **Supplementary Figure S7:** The final genetic map for the B. decumbens CIAT 606 (cv. Basilisk) progenitor of the interspecific population included 4,427 markers placed at LOD 10 in 18 linkage groups.
- **Supplementary Figure S8:** RNA-seq from stem and root tissue samples extracted from the *B. decumbens* and *B. ruziziensis* progenitors. We also incorporated a reanalysis of public RNA-seq data (PRJNA314352) from *B. decumbens* var. Basilisks roots.
- **Supplementary Figure S9:** Enrichment analysis of the "Molecular function" GO terms overrepresented among differentially expressed upregulated (red) or downregulated (blue) genes in roots in *B. decumbens* CIAT 606 and *B. ruziziensis* BRX 44-02.
- **Supplementary Figure S10:** Enrichment analysis of the "Biological Process" GO terms overrepresented among differentially expressed upregulated (red) or downregulated (blue) genes in roots in *B. decumbens* CIAT 606 and *B. ruziziensis* BRX 44-02.
- **Supplementary Figure S11:** Correlation matrix plot among GO terms based on the DE genes included in each annotation.
- **Supplementary Figure S12:** Comparison the enriched GO Slim terms between *B. decumbens* cv. Basilisk exposed to 200 μM $AlCl_3$ for 72 hours and 8 hours, the latter from the reanalysis of public raw data from Salgado *et al*. 2017.

Supplementary table S1: Root length, diameter and biomass in the *B. decumbens* CIAT 606 and *B. ruziziensis* BRX 44-02 (cv. Basilisk) progenitors after growing for 20 days in control and high 200 µM $AlCl_3$ concentration hydroponic solutions

	Root Length (mm)			Root Tip Diameter (mm)			Root biomass (milligrams)		
	Control	Stress	Ratio	Control	Stress	Ratio	Control	Stress	Ratio
B. decumbens CIAT 606	428	261	0.61	0.29	0.31	0.109	54	34	0.63
B. ruziziensis BRX 44-02	177	72	0.41	0.39	0.46	0.118	30	17	0.55
Mean population	474	212	0.45	0.32	0.38	0.118	64	39	0.61

Supplementary Table S2: Statistics of the intermediate steps, alternative assemblies, final assembly and pseudo-molecules for the *B. ruziziensis* CIAT 26162 genome.

Step	Total length (Mbp)	% Ns	Sequences	N50 (Kbp)
WGS Platanus	712.4	17.45	196,321	17.4
ABySS+SOAP2 (Discarded)	815.4	12.74	268,486	5.5
Pacbio Gapfilling	796.8	11.39	191,540	23.3
Deposit WGS (GCA_003016355)	732.5	10.59	102,579	27.8
Unanchored reference (Sequences over 10Kb)	533.9	11.7	23,076	44.6
Anchored in 9 chrs	525.1	12.18	9	55.88*Mbp

Supplementary Table S3: Classification of the repeat content in the Brachiaria genome.

**Category**	**Superfamily**	**Coverage (bps)**	**Fraction genome***
Class 1 Transposable elements (TEs)	Gypsy	156,824,480	23.9
	Copia	62,486,851	9.5
	Pao	55,071	0.0
	Other LTRs	972,934	0.1
	SINEs	2,939,316	0.4
	LINEs	11,929,645	1.8
		(235,208,297)	(35.8)
Class 2 (DNA) Transposable elements (TEs)	hAT	631,797	0.1
	hAT_Ac	2,534,322	0.4
	hAT_Tag1	1,082,474	0.2
	hAT_Tip100	306,963	0.0
	Harbinger/PIF	9,477,600	1.4
	MULE	7,895,375	1.2
	Stowaway	4,436,872	0.7
	CMC_EnSpm	27,724,058	4.2
	Helitron	1,339,398	0.2
		(55,428,859)	(8.4)
Non TEs	Unclassified TE	40,000,354	6.1
	Simple Repeats	482,035	0.1
	Satellites	2,999,591	0.5
		(43,481,980)	(6.6)
Unclassified TE	Other	335,155	0.1
**TOTAL**		**334,454,291**	**51.0**

*656Mbp after excluding ambiguous nucleotides (Ns)

Supplementary Table S4: Alignment of the transcripts and proteins from five sequenced species in the Panicoideae subfamily [foxtail millet (*Setaria italica*), green foxtail (*Setaria viridis* (L.) Beauv.), *Panicum halli* Vasey, switchgrass (*Panicum virgatum* L.), and maize (*Zea mays* L.)], in the *Brachiaria ruziziensis* genome with a minimum identify of 70 %. Transcripts (longest one per gene) were aligned with GMAP and proteins were aligned with Exonerate. Sequences were obtained from Phytozome v.12 or Ensembl (v.284) in the case of maize.

	**Species**	**Total**	**PID>70%**		**PID>70% & PCOV>50%**	
**Transcripts**	***S. italica***	43,001	37,449	87.1	29,534	68.7
	S. viridis	47,205	36,372	77.1	23,110	49
	P. halli	49,852	40,818	81.9	31,599	63.4
	P. virgatum	91,838				
	Z. mays	88,760	58,312	65.7	36,642	41.3
**Proteins**	***S. italica***	43,001	34,749	80.8	29,975	69.7
	S. viridis	47,205	33,157	70.2	27,953	59.2
	P. halli	49,852	37,516	75.3	32,753	65.7
	P. virgatum	91,838				
	Z. mays	88,760	54,091	60.9	45,951	51.8

Supplementary Table S5: EggNOG clusters in six sequenced species in the Panicoideae subfamily, *B. ruziziensis*, foxtail millet (*Setaria italica*), green foxtail (*Setaria viridis* (L.) Beauv.), *Panicum halli* Vasey, switchgrass (*Panicum virgatum* L.), and maize (*Zea mays* L.), classified by number of proteins per cluster.

	P. virgatum		*S. italica*		*S.viridis*		*P. halli*		*B. ruziziensis*		*Z. mays*	
**GENES**	**Num**	**%**	**Num**	**%**	**Num**	**%**	**Num**	**%**	**Num**	**%**	**Num**	**%**
**1**	1897	8.4	18629	84.4	18508	83.4	18413	86.2	12572	66.7	14369	70.3
**2**	11955	53.1	2432	11.0	2534	11.4	2176	10.2	3908	20.7	4250	20.8
**3**	4602	20.5	559	2.5	630	2.8	464	2.2	1164	6.2	1040	5.1
**4**	1808	8.0	193	0.9	235	1.1	157	0.7	472	2.5	381	1.9
**>4**	2237	9.9	263	1.2	285	1.3	163	0.8	733	3.9	411	2.0
**total**	22499		22076		22192		21373		18849		20451	

Supplementary Table S6: Peak and interval positions for the identified QTLs, as well as corresponding *S. italica* chromosome.

Trait*	LG	Peak marker	Peak Position (cM)	Position interval (cM)	Marker interval	LOD	R2	additive effect	Si*
RLA	1	scaf_70183_123	12.65	5.22 - 31.250	scaf_245_124360 - scaf_1729_44015	4.81	13.6	-22.31	8
RLC	1	scaf_2065_28271	26.027	5.22 - 28.893	scaf_1787_41196 - scaf_3809_36896	5.78	16.1	-45.05	8
RRL	3	scaf_1152_23964	96.802	88.62- 98.851	scaf_2718_14282 - scaf_5425_26287	4.75	13.4	3.12	7
RBA	1	scaf_1948_47501	5.22	5.22 - 32.481	scaf_245_124360 - scaf_1218_39495	5.14	14.4	-0.003	8
RBC	1	scaf_18010_6509	25.797	17.162 - 28.893	scaf_7830_644 - scaf_3809_36896	5.25	14.7	-0.004	8
RRD	3	scaf_14238_7306	79.738	79.738 - 83.853	scaf_14238_7306 - scaf_298_25199	4.02	11.5	-2.36	7
RRD	4	scaf_11042_5202	50.423	49.12 - 62.127	scaf_1413_25183 - scaf_1181_29646	4.54	12.8	2.48	3

*Si: *Setaria italica* chromosome.

RLA: Root length in Al^{3+} stress; RLC: Root length in control; RRD: Relative root length ratio (stress/control); RB: Root biomass; RD: Root tip diameter.

Supplementary Table S7: Enrichment analysis of the GO SLIM terms over-represented among DE genes in B. ruziziensis BRX 44-02 (Bruz), B. decumbens CIAT 606 (cv. Basilisk) (Bdec), or PRJNA314352 from Salgado *et al*. (2017).

GO term	MOLEC. FUNC.	Bdec CIAT 606			Bruz BRX 44-02			Salgado *et al*, 2017		
		Pval	REG	GENES	Pval	REG	GENES	Pval	REG	GENES
GO:0003723	RNA binding (3723)	0.05709	down	14	0.40064	down	40	0.988	down	3
GO:0003729	mRNA binding (3729)	0.13999	down	2	0.00358	down	8	0.419	down	1
GO:0003735	structural constituent of ribosome (3735)	5.5E-12	down	24	1E-30	down	112	0.93632	up	5
GO:0005198	structural molecule activity (5198)	0.19102	up	4	0.02756	down	120	0.583	down	2
GO:0008092	cytoskeletal protein binding (8092)	0.58493	down	1	0.25288	down	5	0.035	down	3
GO:0008134	transcription factor binding (8134)	0.0312	up	3	0.85829	down	1	1	0	0
GO:0008289	lipid binding (8289)	0.09253	up	5	0.21271	down	9	0.26684	up	5
GO:0008565	protein transporter activity (8565)	0.08093	up	3	0.14489	down	5	0.446	down	1
GO:0016491	oxidoreductase activity (16491)	0.00034	down	40	6.8E-09	down	143	0.00081	up	69
GO:0016757	glycosil transferase (16757)	0.63379	up	7	0.0438	up	22	0.20773	up	14
GO:0016765	alkyl transferase (16765)	0.46513	down	3	0.03894	down	16	0.000019	up	17
GO:0016798	glycosyl hydrolase (16798)	0.00084	up	18	0.0002	down	39	0.00041	up	24
GO:0016829	lyase activity (16829)	0.01422	up	10	0.16411	down	16	2E-12	up	30
GO:0016853	isomerase activity (16853)	0.43965	down	4	0.00077	down	26	0.302	down	4
GO:0016874	ligase activity (16874)	0.11268	up	8	0.1587	up	13	0.0492	up	12
GO:0019843	rRNA binding (19843)	0.04791	down	3	0.0000021	down	14	0.81806	up	1
GO:0019899	enzyme binding (19899)	0.00567	down	8	0.90566	down	7	0.65	down	2
GO:0022857	transmembrane transporter activity (22857)	0.00000018	up	44	0.00000031	up	70	0.00000015	up	57
GO:0030234	enzyme regulator activity (30234)	0.58307	down	3	0.00354	down	22	0.09403	up	10
GO:0030674	protein binding bridging (30674)	1	0	0	0.06958	down	2	1	0	0
GO:0043167	ion binding (43167)	0.23666	up	44	0.0042	up	100	0.261	down	26
GO:0051082	unfolded protein binding (51082)	0.09328	down	2	0.32559	down	3	1	0	0

REG: Either up-regulated (up) or down-regulated (down)

Supplementary Table S7 -Cont.-

GO term	BIOLOG. PROCESS.	Bdec CIAT 606			Bruz BRX 44-02			Salgado *et al*, 2017		
		Pval	REG	GENES	Pval	REG	GENES	Pval	REG	GENES
GO:0005975	carbohydrate metabolic process (5975)	0.000087	1	21	0.00518	-1	42	0.00193	1	24
GO:0006091	generation of precursor metabolites (6091)	0.44019	-1	4	0.0175	1	15	0.0000086	-1	13
GO:0006397	mRNA processing (6397)	0.06748	-1	5	0.6105	1	5	0.91865	1	2
GO:0006412	translation (6412)	0.0002	-1	18	1E-30	-1	103	0.90928	1	9
GO:0006457	protein folding (6457)	0.17529	-1	3	0.01588	-1	12	0.7229	-1	1
GO:0006464	cellular protein modification process (6464)	0.03977	-1	23	0.9702	1	28	0.3059	-1	16
GO:0006520	cellular amino acid metabolic process (6520)	0.4388	1	6	0.4677	1	11	0.03173	1	14
GO:0006629	lipid metabolic process (6629)	0.00645	-1	14	0.00613	-1	41	0.02375	1	20
GO:0006810	transport (6810)	0.0114	1	35	0.03057	-1	83	0.6354	-1	12
GO:0006913	nucleocytoplasmic transport (6913)	0.0052	1	7	0.232	1	6	0.7814	-1	1
GO:0006914	autophagy (6914)	1	-1	0	0.1436	1	3	0.0812	1	3
GO:0006950	response to stress (6950)	0.591	1	14	0.4765	1	29	0.0521	-1	16
GO:0007005	mitochondrion organization (7005)	0.08128	-1	3	0.02374	-1	9	0.57158	1	2
GO:0007010	cytoskeleton organization (7010)	0.76433	-1	1	0.13705	-1	9	0.0371	-1	4
GO:0007155	cell adhesion (7155)	1	-1	0	0.0699	1	1	1	-1	0
GO:0007165	signal transduction (7165)	0.1634	1	15	0.0845	1	29	0.09511	1	23
GO:0009056	catabolic process (9056)	0.13519	-1	17	0.20889	-1	58	1.2E-09	1	58
GO:0009058	biosynthetic process (9058)	0.09869	-1	52	0.0267	1	95	0.1459	-1	35
GO:0019748	secondary metabolic process (19748)	0.011	1	13	0.0066	1	22	0.00064	1	21
GO:0022618	ribonucleoprotein complex assembly (22618)	0.00041	-1	8	0.000012	-1	21	0.85132	1	2
GO:0030154	cell differentiation (30154)	0.5706	1	1	0.05932	-1	6	0.4517	-1	1
GO:0030198	extracellular matrix organization (30198)	1	-1	0	0.0699	1	1	0.05441	1	1
GO:0042592	homeostatic process (42592)	0.3365	1	7	0.0526	1	17	0.00568	1	17
GO:0044281	small molecule metabolic process (44281)	0.04068	-1	19	0.00017	-1	75	3.6E-09	1	60
GO:0048856	anatomical structure development (48856)	0.4292	1	13	0.0384	1	29	0.8382	-1	7
GO:0051186	cofactor metabolic process (51186)	0.1532	-1	6	0.01469	-1	24	0.000004	1	21
GO:0051301	cell division (51301)	1	-1	0	0.00458	-1	7	1	0	0
GO:0055085	transmembrane transport (55085)	0.14685	-1	3	0.30994	-1	7	0.01203	1	7
GO:0071554	cell wall organization or biogenesis (71554)	0.0000041	1	17	2.8E-14	-1	51	0.01151	1	14

Supplementary Figure S1: 31mer frequency analysis comparing the short-reads assemblies produced with *Platanus assembler* or the alternative approach using the combination of ABySS for isotigs assembly and SOAP2 for scaffolding. The area under the curve of the Kmer spectra has been coloured according to the number of times that such K-mers appear in the assembly: none in back, once in red, twice in orange, etc.

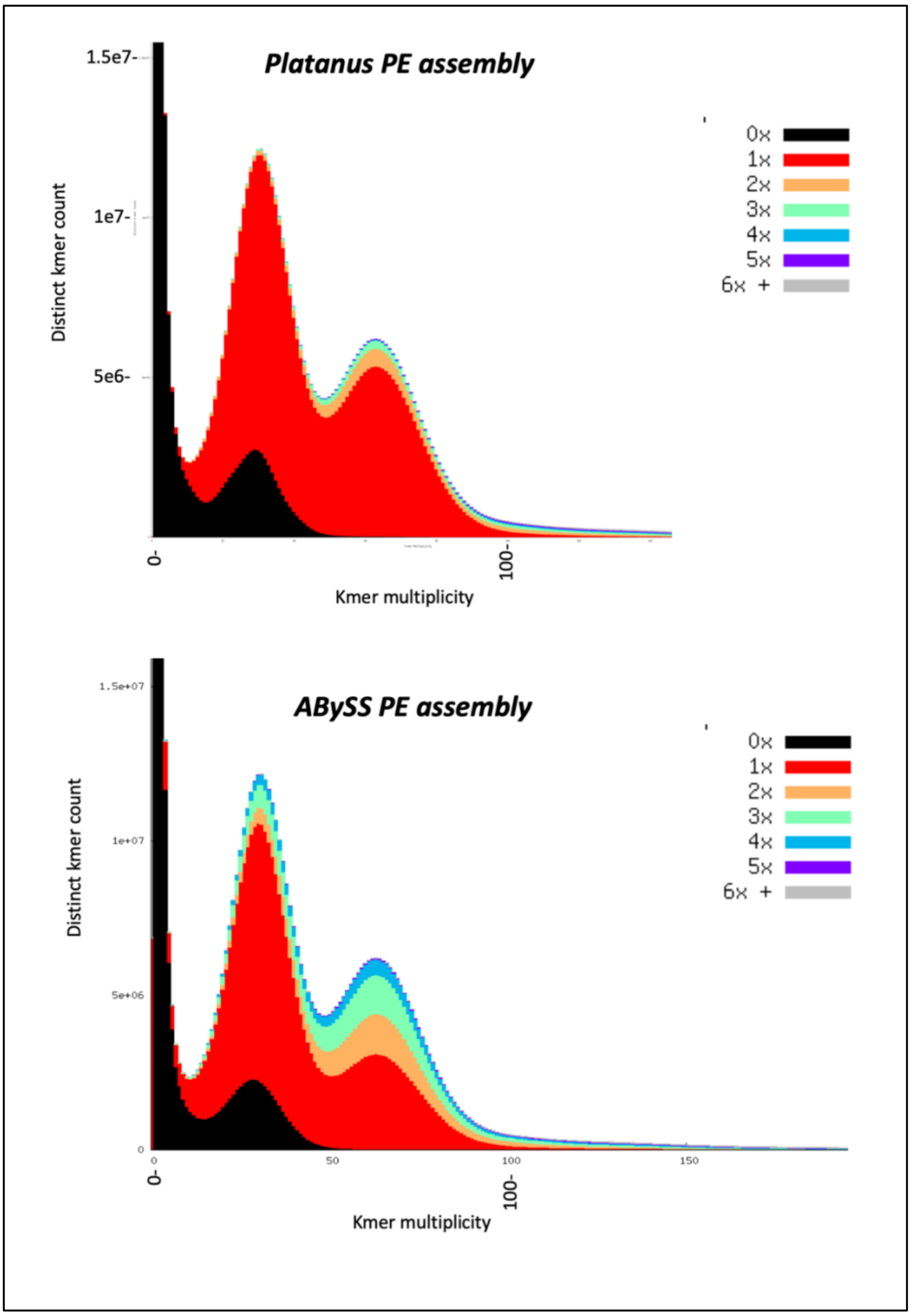

Supplementary Figure S2: Divergence (Kimura) rates between the flanking tails in each *Gypsy* and *Copia* LTR duplication events in the *Brachiaria* genome.

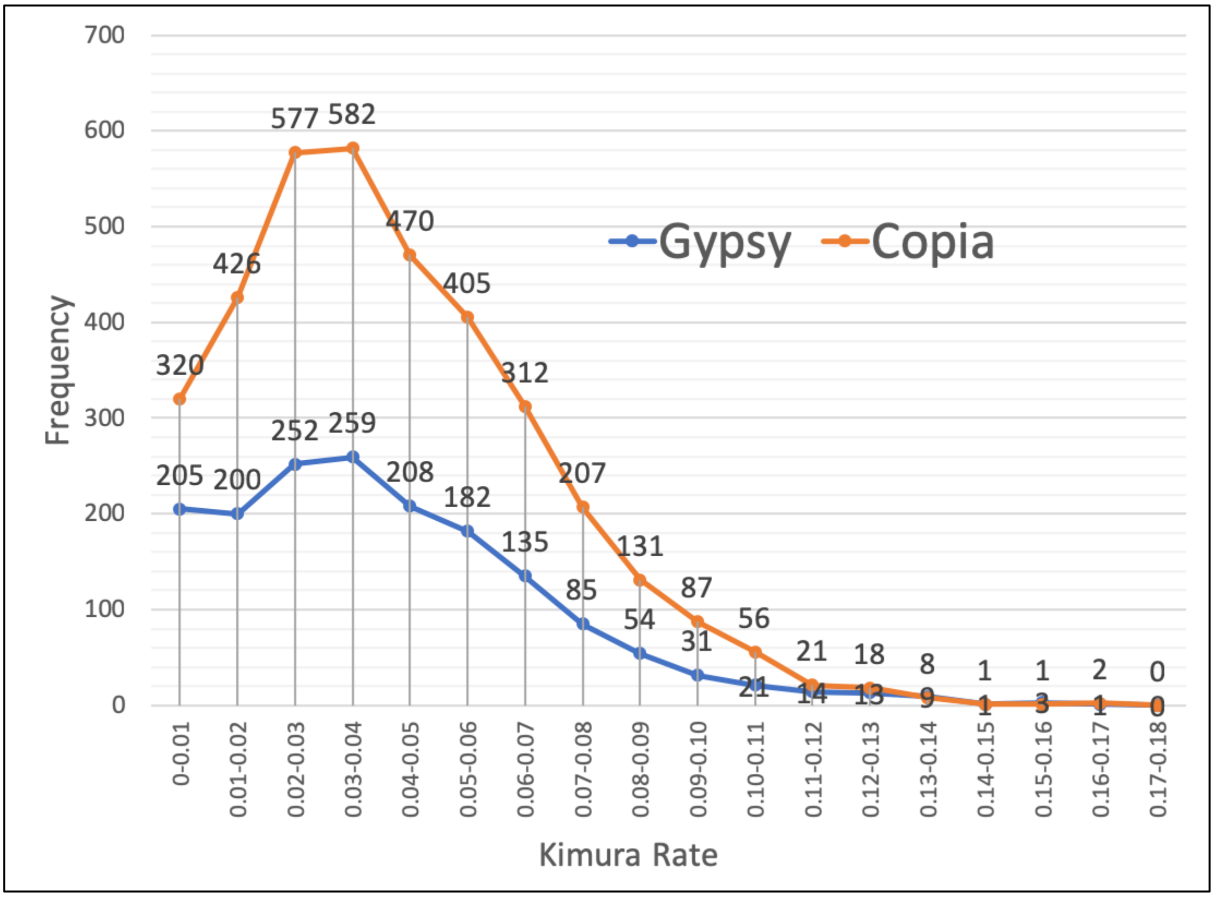

Supplementary Figure S3: Species of the top Blastp hit for each 35,982 of the coding transcripts which had a homologous protein in the NCBI non-redundant (nr) database.

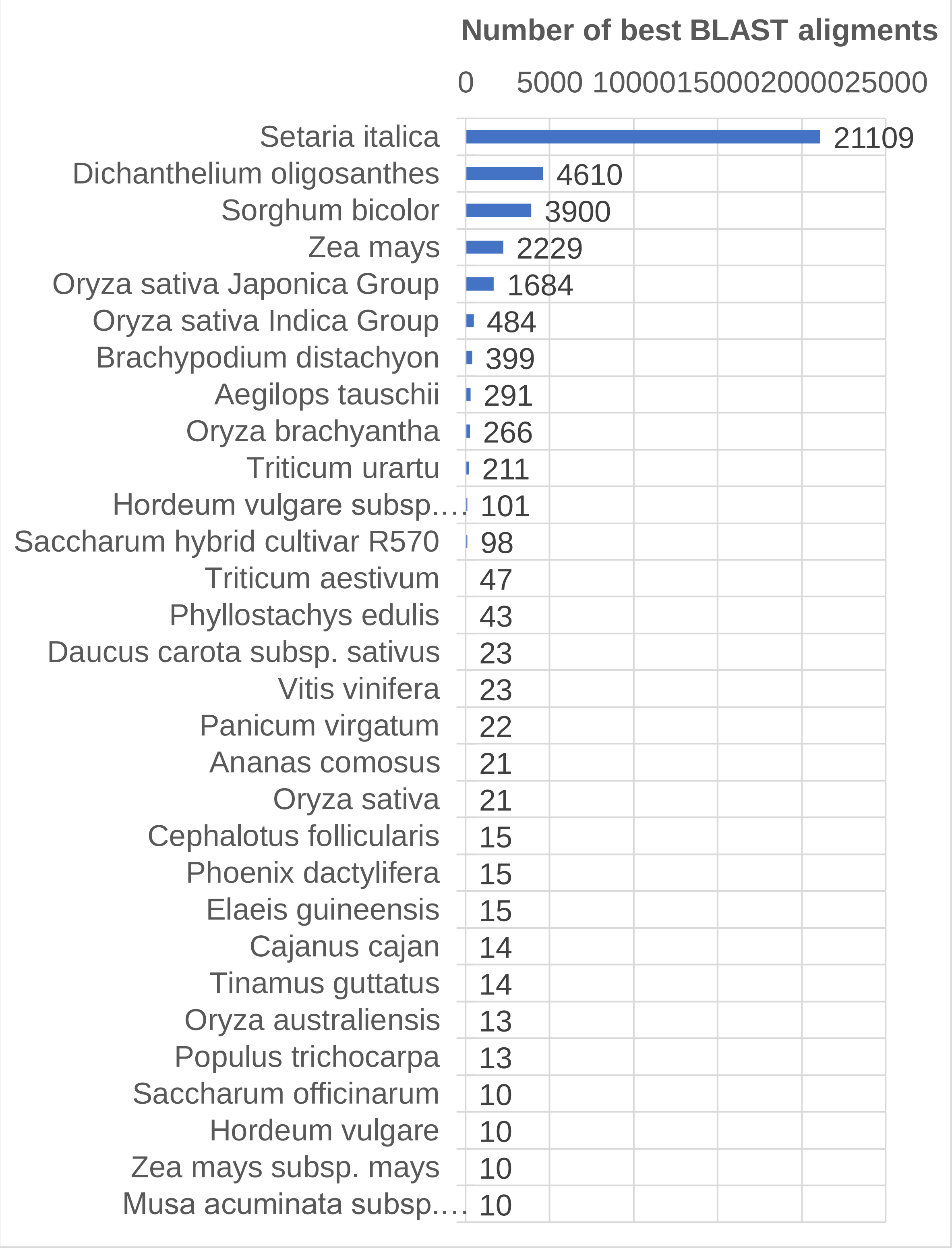

Supplementary Figure S4: Shared eggnog clusters of proteins among *Brachiaria ruziziensis* (Bruz), foxtail millet [*S. italica* (Sita)], *S. viridis* (Svir), maize [*Z. mays* (Zmays)], *Panicum halli* (Phal) and switchgrass [*P. virgatum* (Pvir)]. The "UpSet" plot format provides an efficient way to visualize the intersections (columns) of six species (Rows).

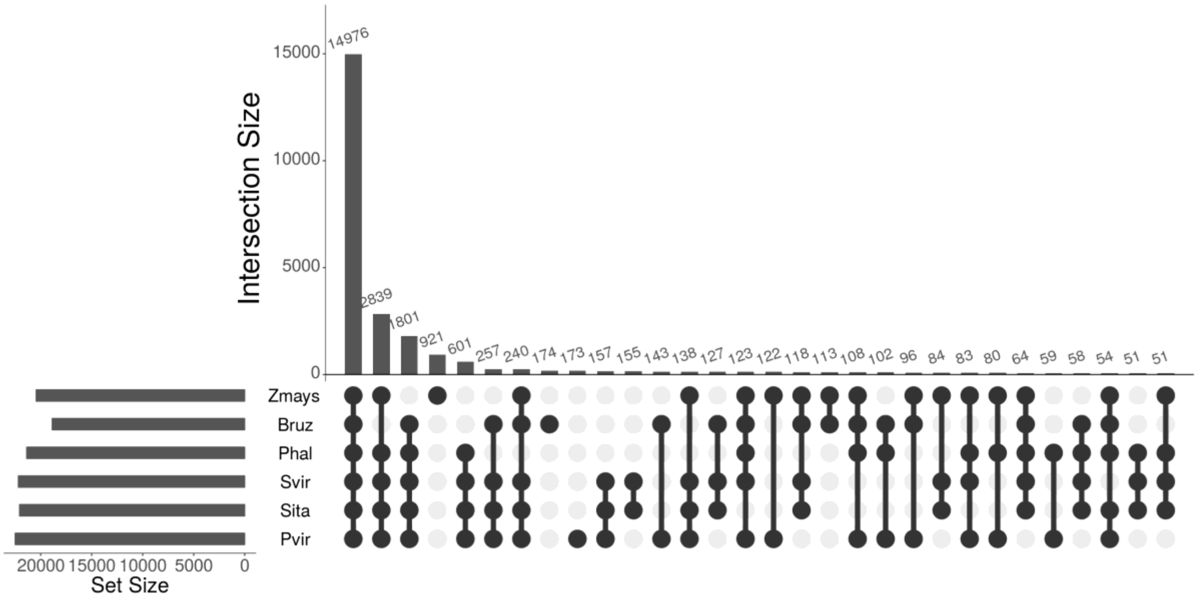

Supplementary Figure S5: Kimura rates between homologous gene pairs between *B. ruziziensis* and sequenced relatives including foxtail millet [*S. italica* (Sita)], *S. viridis* (Svir), maize [*Z. mays* (Zmays)], and *P. halli* (Phal)]. Gene pairs were build based on eggNOG clusters.

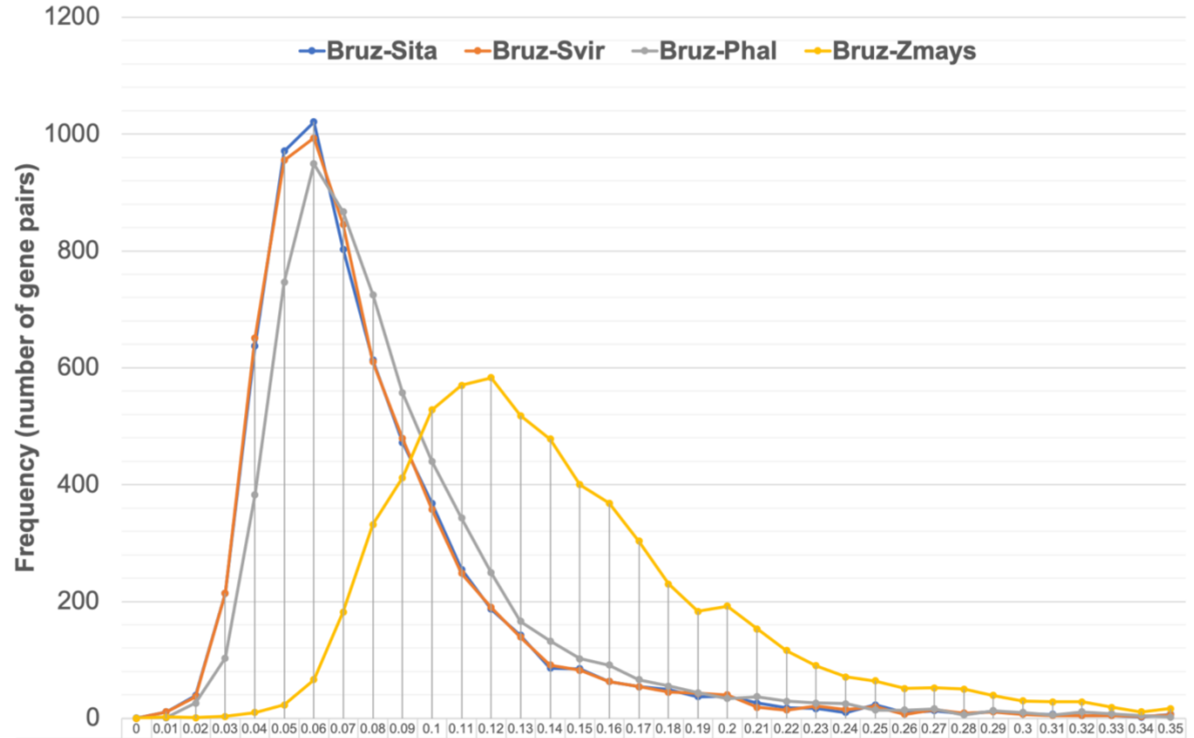

	0	0.01	0.02	0.03	0.04	0.05	0.06	0.07	0.08	0.09	0.1	0.11	0.12	0.13	0.14	0.15	0.16	0.17
Bruz-Sita	0	11	39	214	637	971	1021	803	613	472	368	255	187	142	86	85	63	54
Bruz-Svir	0	11	37	214	651	955	993	845	610	479	358	248	190	139	91	82	63	54
Bruz-Phal	1	1	26	103	383	747	949	867	725	557	440	343	249	166	132	102	91	66
Bruz-Zmays	0	2	1	3	10	23	66	182	332	412	528	570	583	518	478	400	368	303

	0.18	0.19	0.2	0.21	0.22	0.23	0.24	0.25	0.26	0.27	0.28	0.29	0.3	0.31	0.32	0.33	0.34	0.35
Bruz-Sita	49	37	38	26	18	17	10	23	10	13	8	12	8	7	5	5	2	7
Bruz-Svir	45	43	40	19	14	21	15	20	7	15	9	11	7	5	5	5	3	6
Bruz-Phal	55	44	34	37	29	26	25	14	14	16	6	13	10	6	11	8	5	2
Bruz-Zmays	230	183	192	153	116	90	71	64	51	52	50	39	30	28	28	19	11	17

Supplementary Figure S6: Phylogenetic tree based on nucleotide divergence rate between sequences in the same eggnog cluster from *B. ruziziensis* and sequenced relatives including foxtail millet [*S. italica* (Sita)], *S. viridis* (Svir), maize [*Z. mays* (Zmays)], and *P. halli* (Phal)].

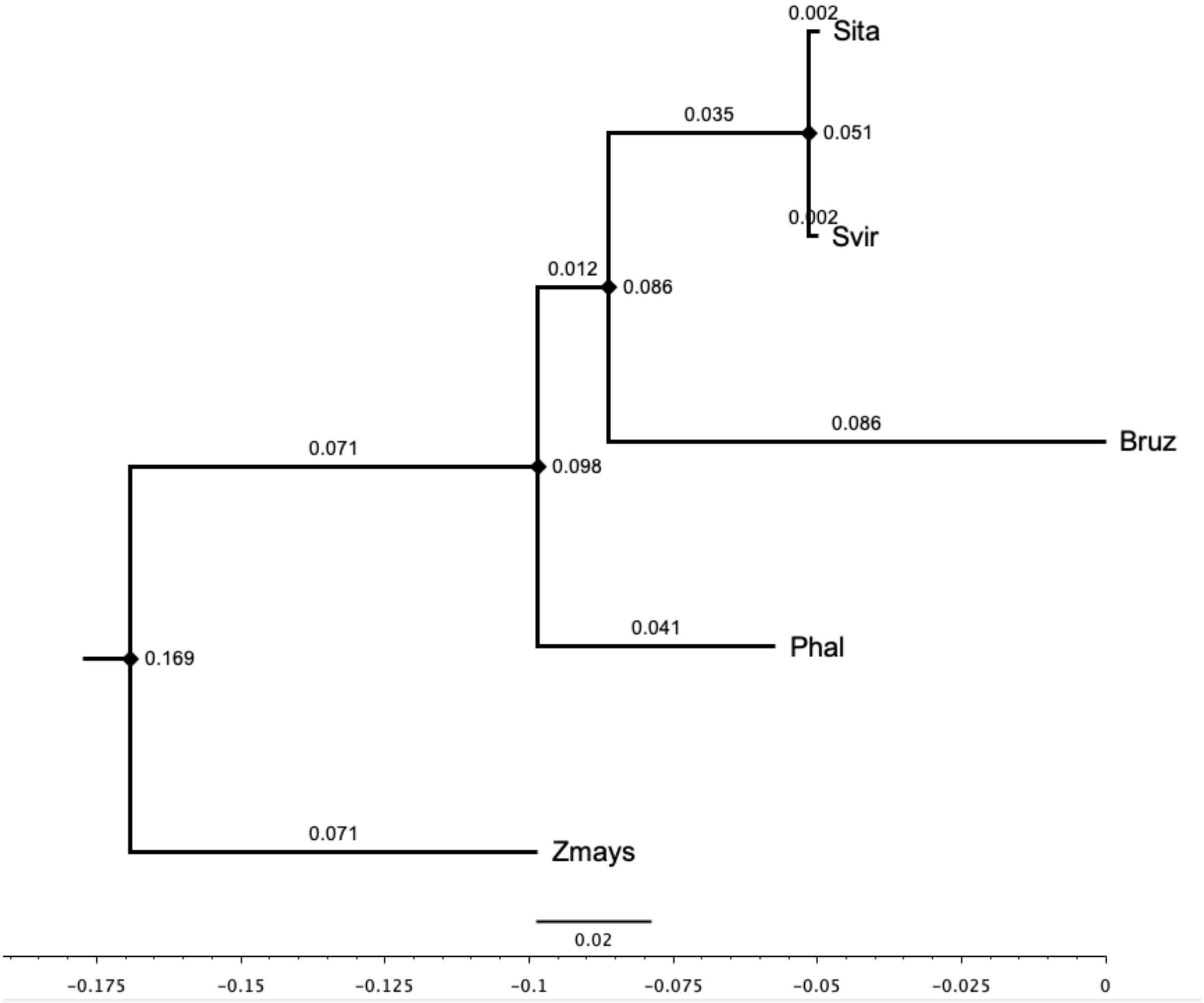

Supplementary Figure S7: The final genetic map for the B. decumbens CIAT 606 (cv. Basilisk) progenitor of the interspecific population included 4,427 markers placed at LOD 10 in 18 linkage groups

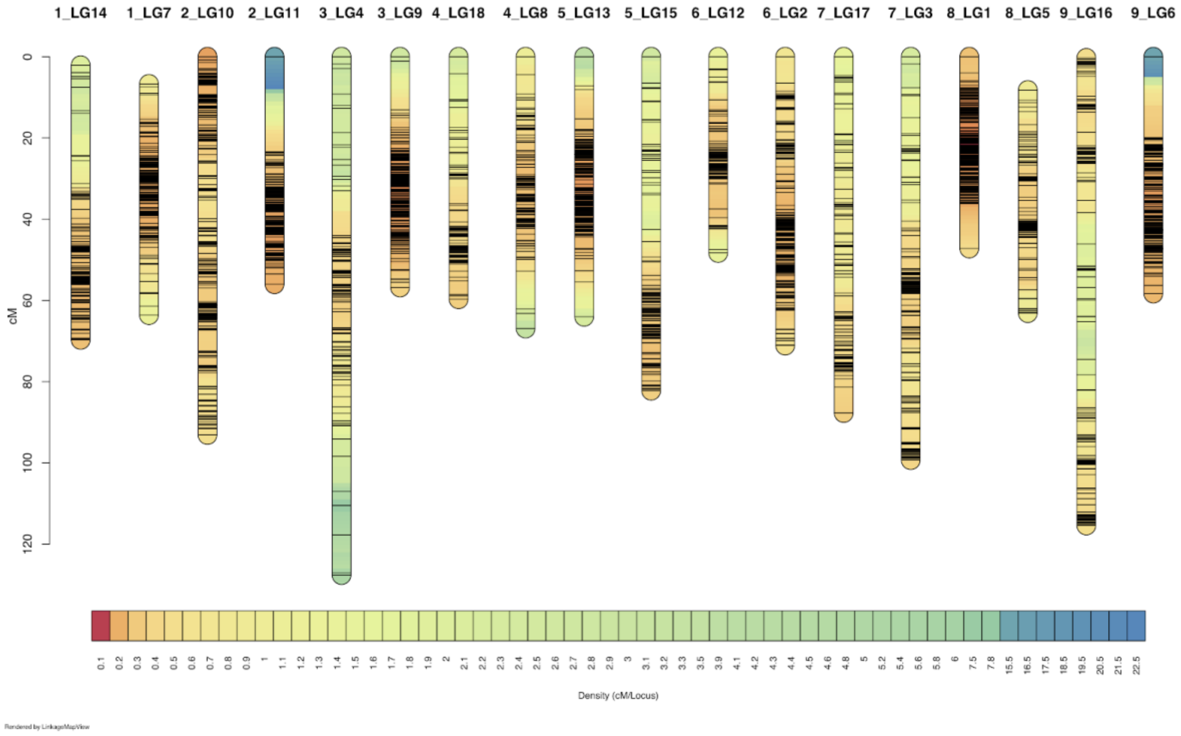

Supplementary Figure S8: RNA-seq from stem and root tissue samples extracted from the *B. decumbens* and *B. ruziziensis* progenitors. We also incorporated a reference-based reanalysis of public RNA-seq data (PRJNA314352) from *B. decumbens* var. Basilisks roots (Salgado et al., 2017, Plant Growth Regulation, 83,1:157-170). When the normalised counts for all the genes were used to cluster the samples, these clusters firstly grouped by tissue, secondly by genotype, and thirdly by treatment.

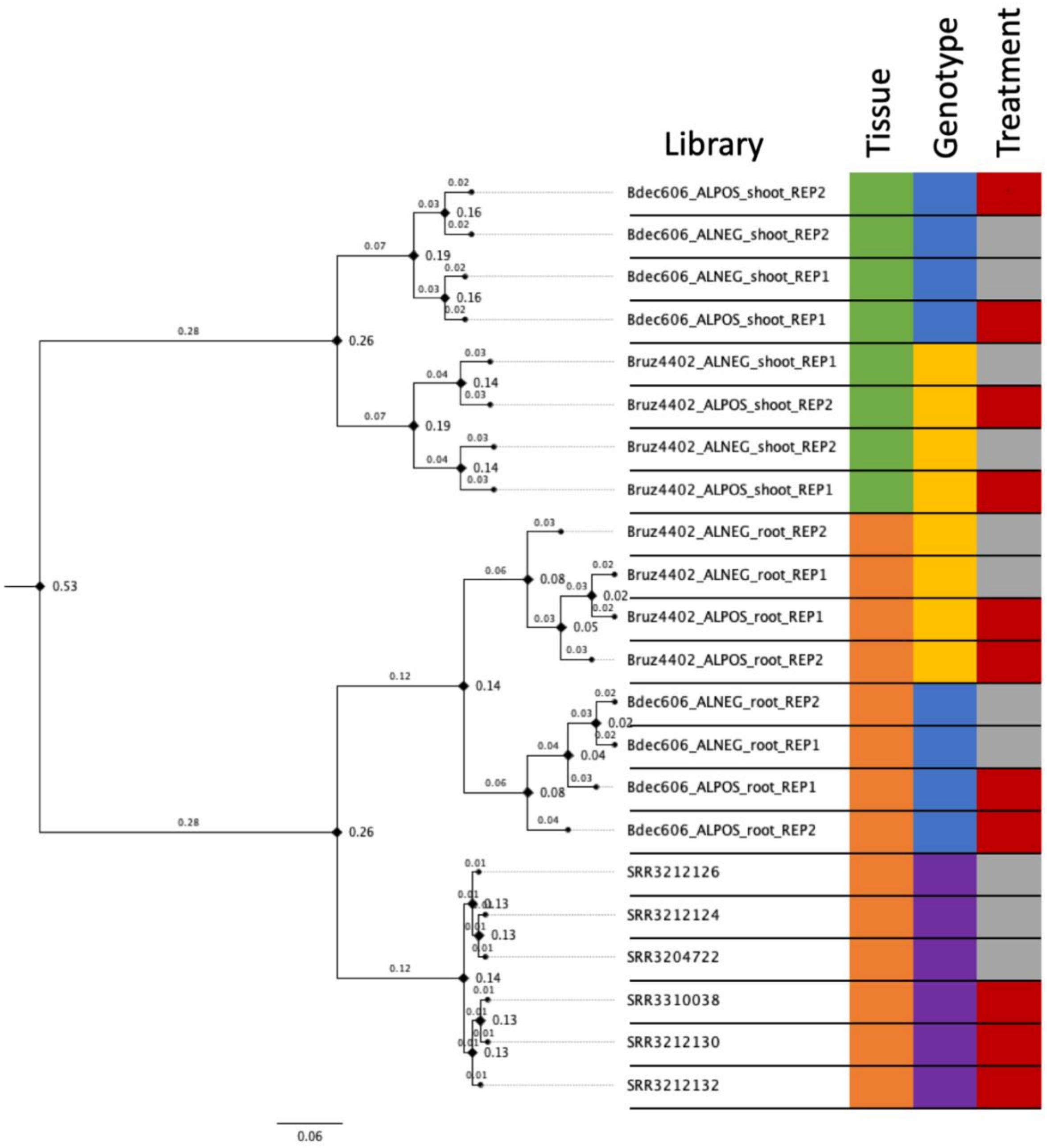

Supplementary Figure S9: Enrichment analysis of the "Molecular function" GO terms overrepresented among differentially expressed upregulated (red) or downregulated (blue) genes in roots in *B. decumbens* CIAT 606 and *B. ruziziensis* BRX 44-02.

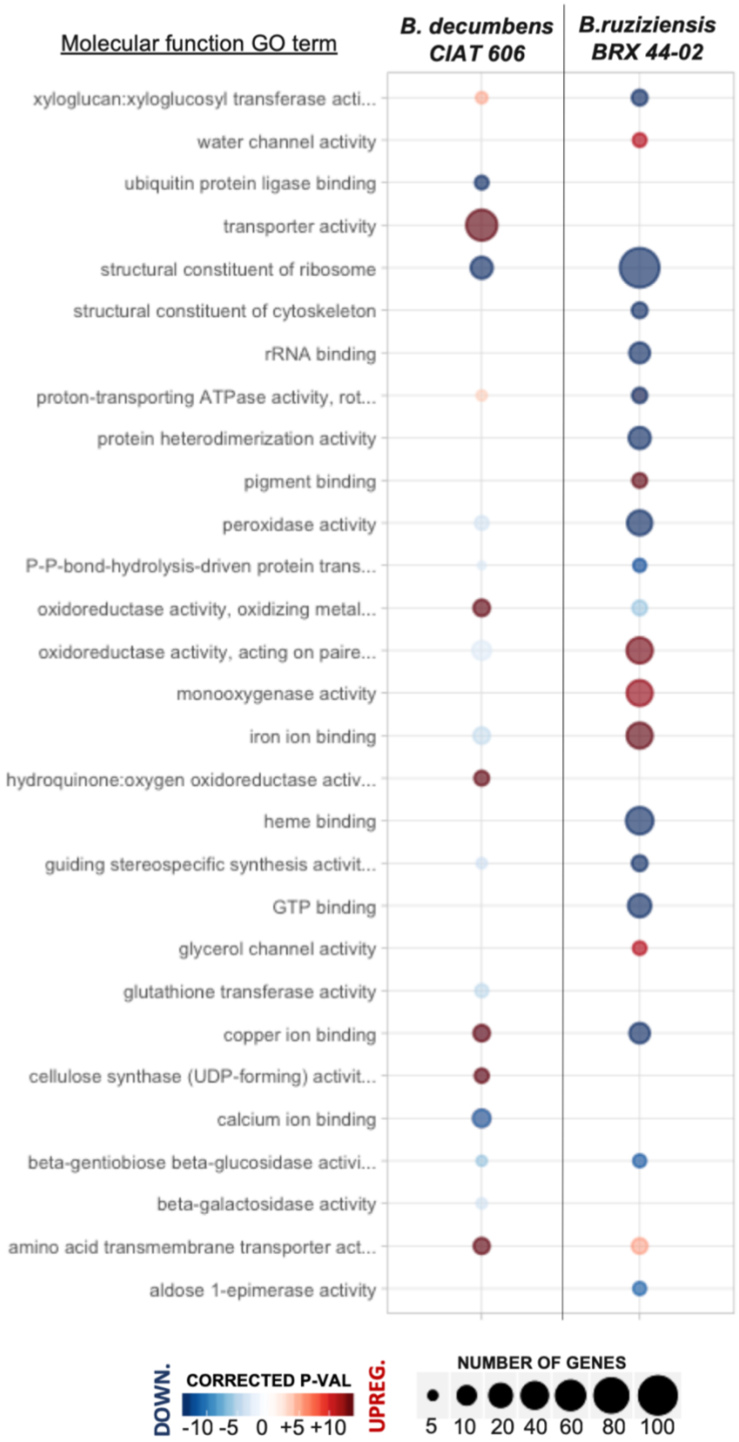

Supplementary Figure S10: Enrichment analysis of the "Biological Process" GO terms overrepresented among differentially expressed upregulated (red) or downregulated (blue) genes in roots in *B. decumbens* CIAT 606 and *B. ruziziensis* BRX 44-02.

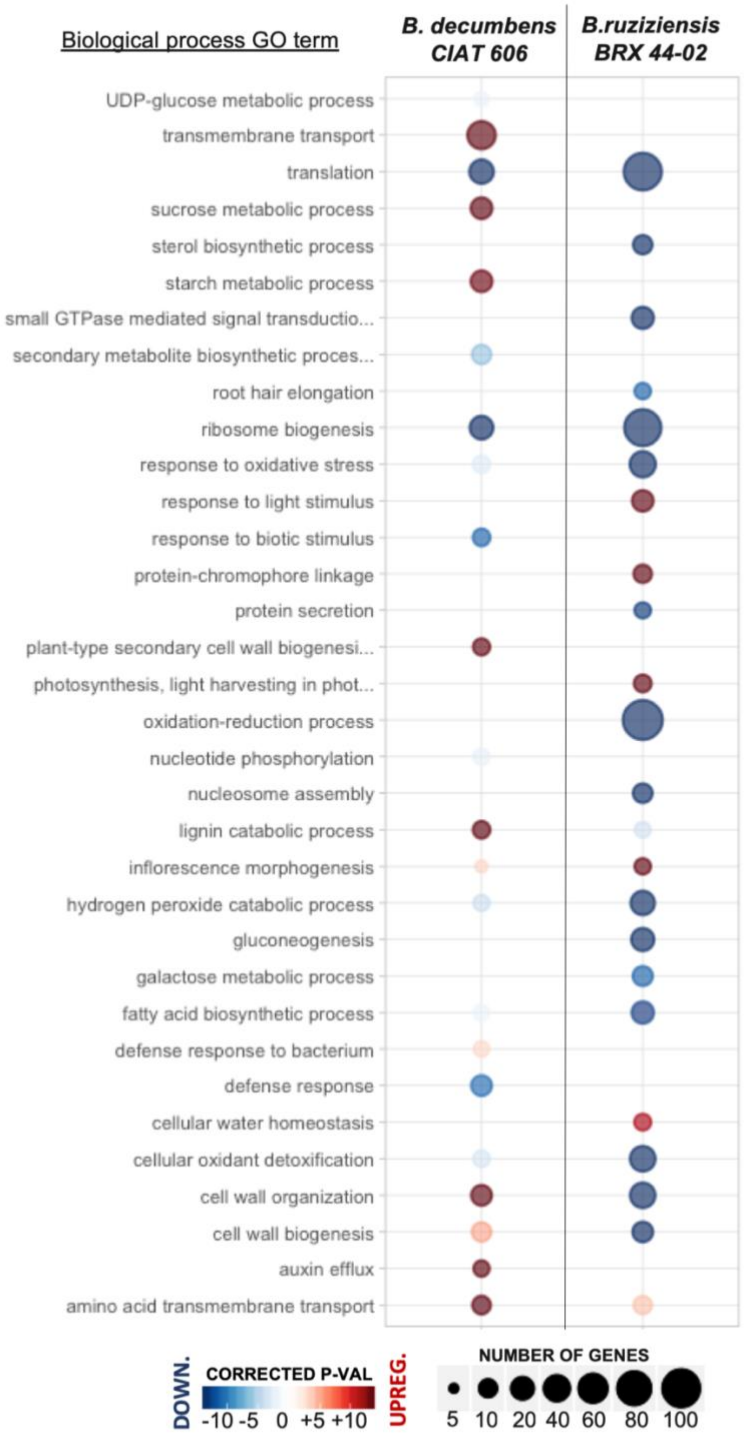

Supplementary Figure S11: Correlation matrix plot among GO terms based on the DE genes included in each annotation.

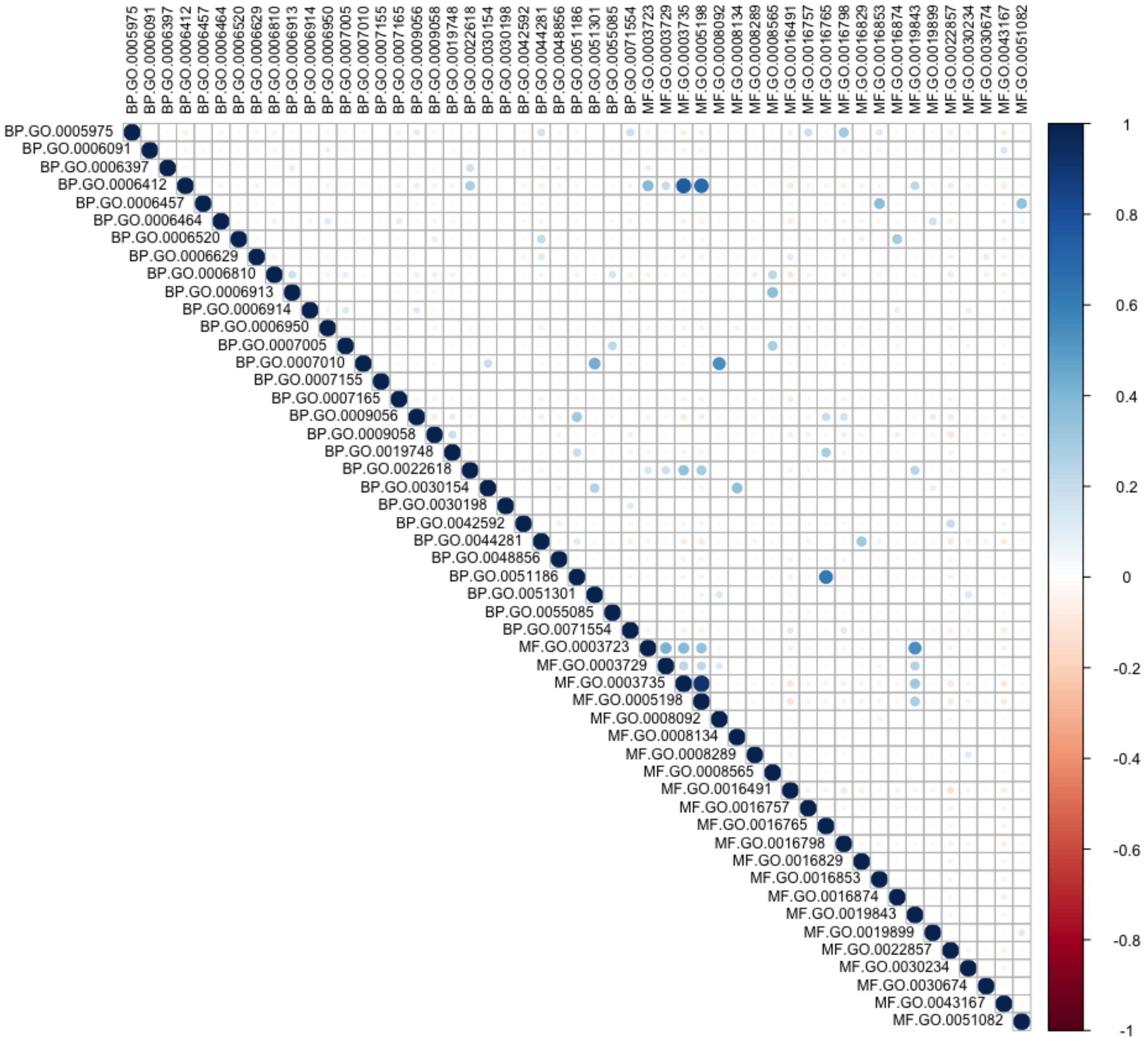

Supplementary Figure S12: Comparison the enriched GO Slim terms between *B. decumbens* cv. Basilisk exposed to 200 μM $AlCl_3$ for 72 hours and 8 hours, the latter from the reanalysis of public raw data from Salgado *et al*. 2017.

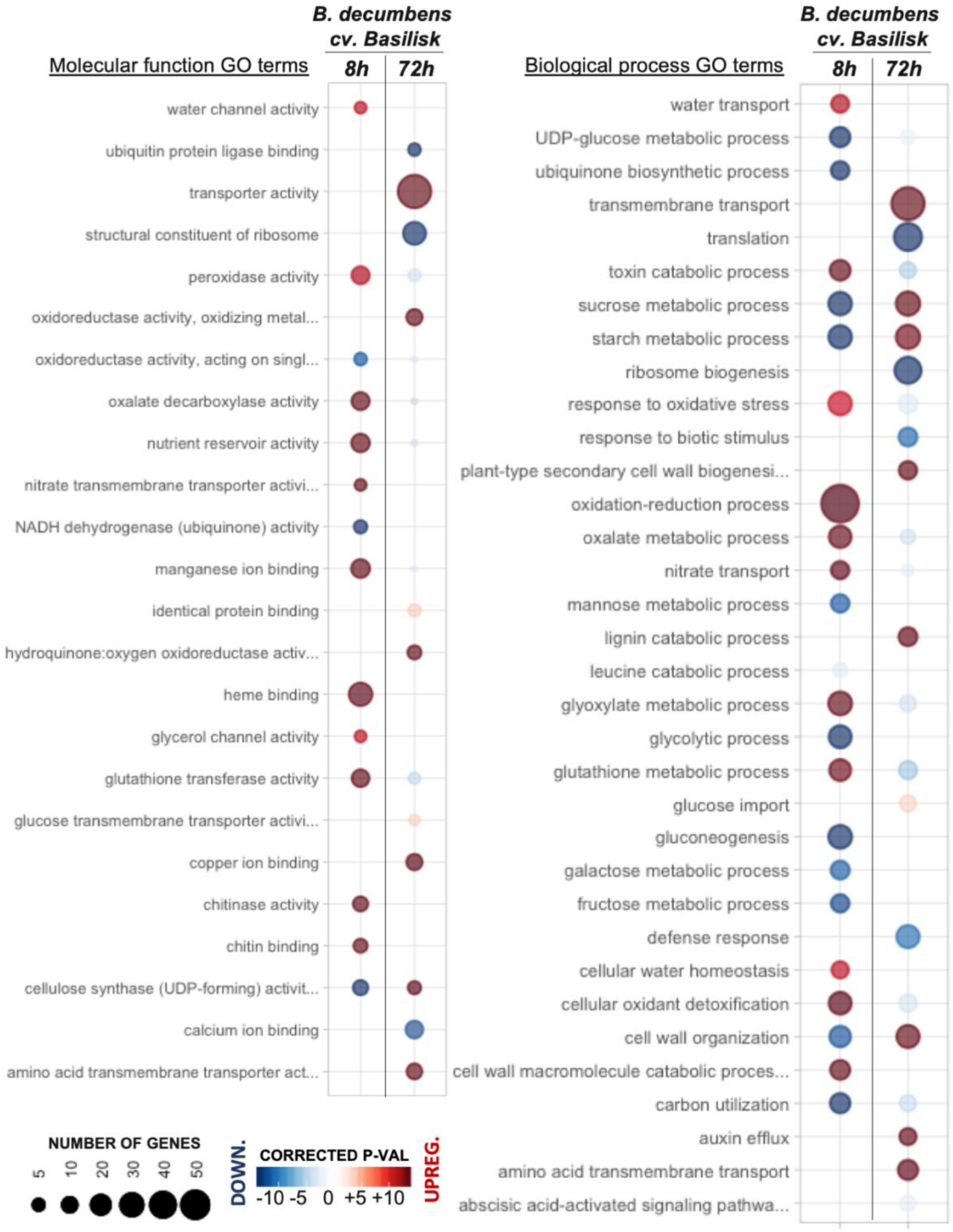

Supplement: eraa469_suppl_Supplementary_Materials [file eraa469_suppl_supplementary_materials.pdf]
